# Effects of Aggregation on Blood Sedimentation and Conductivity

**DOI:** 10.1371/journal.pone.0129337

**Published:** 2015-06-05

**Authors:** Alexander Zhbanov, Sung Yang

**Affiliations:** 1 Department of Medical System Engineering, Gwangju Institute of Science and Technology, Gwangju, Republic of Korea; 2 School of Mechatronics, Gwangju Institute of Science and Technology, Gwangju, Republic of Korea; Institut national de la santé et de la recherche médicale—Institut Cochin, FRANCE

## Abstract

The erythrocyte sedimentation rate (ESR) test has been used for over a century. The Westergren method is routinely used in a variety of clinics. However, the mechanism of erythrocyte sedimentation remains unclear, and the 60 min required for the test seems excessive. We investigated the effects of cell aggregation during blood sedimentation and electrical conductivity at different hematocrits. A sample of blood was drop cast into a small chamber with two planar electrodes placed on the bottom. The measured blood conductivity increased slightly during the first minute and decreased thereafter. We explored various methods of enhancing or retarding the erythrocyte aggregation. Using experimental measurements and theoretical calculations, we show that the initial increase in blood conductivity was indeed caused by aggregation, while the subsequent decrease in conductivity resulted from the deposition of erythrocytes. We present a method for calculating blood conductivity based on effective medium theory. Erythrocytes are modeled as conducting spheroids surrounded by a thin insulating membrane. A digital camera was used to investigate the erythrocyte sedimentation behavior and the distribution of the cell volume fraction in a capillary tube. Experimental observations and theoretical estimations of the settling velocity are provided. We experimentally demonstrate that the disaggregated cells settle much slower than the aggregated cells. We show that our method of measuring the electrical conductivity credibly reflected the ESR. The method was very sensitive to the initial stage of aggregation and sedimentation, while the sedimentation curve for the Westergren ESR test has a very mild slope in the initial time. We tested our method for rapid estimation of the Westergren ESR. We show a correlation between our method of measuring changes in blood conductivity and standard Westergren ESR method. In the future, our method could be examined as a potential means of accelerating ESR tests in clinical practice.

## Introduction

The determination of the erythrocyte sedimentation rate (ESR) is a useful hematological test because it provides a measure of the patient’s inflammatory or acute-phase response. The Polish physician Edmund Faustyn Biernacki invented a method of measuring the ESR in 1897 [1]. Later, similar methods were also reported by Robert Sanno Fåhræus in 1918 [2] and Alf Vilhelm Albertsson Westergren in 1921 [3]. The Westergren ESR method is simple and inexpensive; it rapidly entered widespread use throughout the world.

In the Westergren method [4], venous blood is mixed 4:1 with sodium citrate and collected in a glass or plastic tube with a minimum sedimentation scale of 200 mm and a minimum bore of 2.55 mm. The tube is fixed vertically in a Westergren stand at room temperature. At the end of 1 h, the distance from the lowest point of the surface meniscus to the top level of the red cell sediment is recorded as the ESR in mm/h.

Many research groups have studied the kinetics of erythrocyte sedimentation [5–8]. The fall of the interface at the top of the cell column follows a sigmoid shaped curve in time, which is the erythrocyte sedimentation curve. This curve consists of three phases: an initial phase, which includes the aggregation and acceleration of erythrocyte sedimentation; a long phase, during which there is a constant rate of fall; and a final deceleration phase, which covers the packing of aggregates at the bottom of the tube. Note that the initial phase is defined approximately because the meniscus makes observations difficult. Therefore, the initial phase requires further, more careful study.

The Westergren test has a duration of 1 h, making it significantly longer than other routine automated hematological tests. Research has been undertaken into methods that would allow ESR to be evaluated in a shorter period of time [9–11]. The International Committee for Standardization in Hematology strongly recommends the use of the conventional Westergren method as a means of testing new technologies [4]. Before more rapid methods of assessing ESR can be introduced into clinical practice, the mechanisms of aggregation that influence ESR must be clarified by fundamental research. Pribush *et al*. [12] have critically reviewed recent models of erythrocyte sedimentation.

It has been suggested that measurements of the electrical impedance of the entire blood column could be used to determine the ESR [13]. The resistivity of blood has been shown to be closely correlated to the hematocrit (HCT) (the volume of red blood cells as a percentage of the whole blood volume) [14, 15]. A simple formula for estimating the ESR using the plasma resistance, membrane capacitance, and HCT, has been obtained by linear regression [16]. In addition, more recently, the time dependence of the ESR and conductivity has been investigated by Cha *et al*. [15] and Pribush *et al*. [12, 17–19]. These studies focused on the phenomenon that, as the erythrocytes settle over time, the HCT decreases in the upper region of the blood column.

The changes in conductivity at the bottom of the blood column were first studied in our work [20]. We assume that these measurements best reflect the erythrocyte sedimentation and aggregation. We used a small chamber with two planar electrodes on the bottom. Our experiments showed that the conductivity of the blood in the chamber increased during the first minute of observation. The conductivity then decreased, and continued to do so for more than 2 h. We hypothesized and then demonstrated theoretically that the increase in blood conductivity within the first minute was due to erythrocyte aggregation, while the subsequent decrease in conductivity resulted from the deposition of erythrocytes [20]. If the electrodes had been placed at the top of the column, we would have expected the conductivity to have increased, as has been studied by Cha *et al*. [15] and Pribush *et al*. [12, 17–19]. However, such measurements miss a great deal of information concerning erythrocyte sedimentation.

Erythrocyte aggregation at non-steady flow conditions in the Couette rheometric cell has been studied by Kaliviotis et al. [21]. Electro-rheology was applied in parallel with optical shearing technique to study blood microstructural characteristics. Nevertheless, the character of fluid flow in the Couette cell differs appreciably from the gravitational blood sedimentation.

The aim of this work is two-fold: first, to continue a basic research investigation, through which we sought to estimate the effect of cell aggregation on blood sedimentation and conductivity; and secondly, to suggest a new rapid method for estimation of the Westergren ESR.

We explored various methods of enhancing or retarding the erythrocyte aggregation. Using new experimental measurements in our chamber, we show that the initial increase in blood conductivity was indeed caused by aggregation.

The usual volume fraction of erythrocytes in human blood is about 0.45. Describing sedimentation in such a dense suspension presents considerable challenge, even for disaggregated particles. To the best of our knowledge, the literature does not include experimental data on the profile of the erythrocyte volume fraction in the blood column. We used a digital camera to determine the erythrocyte sedimentation curve, velocity of sedimentation, and volume fraction profile.

Our long-term goal is to determine the relationship between ESR and blood conductivity during aggregation and sedimentation. As a step towards this goal, we have proposed a simplified model of erythrocyte sedimentation.

Our method of measuring the electrical conductivity of blood is very sensitive to the initial stage of sedimentation. We suggest this method for rapid estimation of the Westergren ESR. We show a correlation between the rate of changes in blood conductivity and standard Westergren ESR method. Our method requires 400 s settling time for the test. We believe that, in the longer term, our method could be investigated as a potential means of accelerating ESR tests in clinical practice. For this purpose, we have refined our measuring system into a device that is easy to use and suitable for potential application in point-of-care testing.

## Materials and Methods

### Ethics statement

Blood components of healthy volunteers were obtained from the Korean Red Cross. The Gwangju branch of the Korean Red Cross provided the following separate components for research performed at the Institute of Medical System Engineering at Gwangju Institute of Science and Technology: red blood cells, platelets, white blood cells, and plasma (Reference contract: 20140410-HR-11-02-02). All blood donors provided written informed consent and completed a standard questionnaire in the Gwangju branch of the Korean Red Cross.

### Experimental methods

#### The blood conductivity measuring system

**Fig 1:**
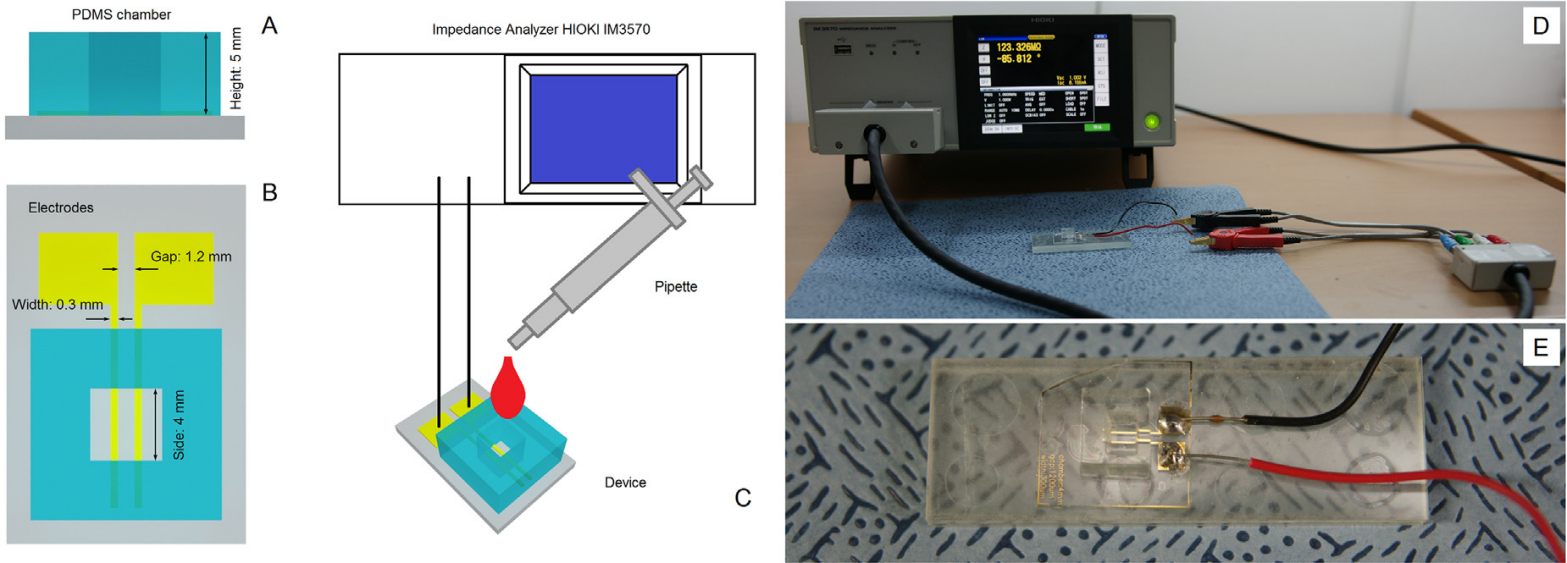
Schematic drawing of the device and experimental setup that were used to measure blood conductivity. (A) Cross-section of the PDMS chamber. (B) Shapes and sizes of the gold-plated electrodes on the bottom of the chamber. (C) The appearance of the measuring system. Blood samples are loaded into the chamber using a pipette. (D) Photograph of the experimental setup. Calibration using air. (E) Photograph of the measuring PDMS chamber with two planar electrodes.

A schematic drawing of the device that we used to measure blood conductivity is shown in Fig 1A. The device is prepared following the conventional Micro-Electro-Mechanical Systems process. It consists of a polydimethylsiloxane (PDMS) chamber with a square cross-section (sides: 4 mm wide and 5 mm deep, as shown in Fig 1A), and two gold-plated two-dimensional planar electrodes that are set 1200 μm apart and each have a width 300 μm (Fig 1B). The portion of the electrode has contact with blood is about 4 mm in length. The planar electrodes were fabricated using a typical lithographic process. The positive photoresist GXR-601 (AZ Electronic Materials, Anseong, Korea) was patterned on a glass substrate sputtered with 300-nm gold and 30-nm chromium for adhesion. After the lithography patterning of the electrodes, the substrate was immersed in gold etchant (Sigma Aldrich, Corp., St. Louis, MO, United States) to remove gold and chromium. The well-like chamber illustrated in Fig 1 was created in a PDMS block using a 4 × 4 mm punch. Subsequently, the glass-bottomed substrate (integrated with the electrodes) was bonded with the PDMS block via an O_2_ plasma process.

The measurement system is shown schematically in Fig 1C. It consists of the device shown in Fig 1A and an impedance analyzer (HIOKI IM3570; HIOKI, Corp., Nagano, Japan). Blood samples are loaded into the chamber using a pipette (Fig 1C). When assessing the performance of the proposed method, we used the same device three times for each sample. The measured resistance of the blood sample depends on the geometrical dimensions of the chamber and the electrode configurations. We used a KCl standard solution of known conductivity (Thermo Fisher Scientific, Beverly, MA, USA) to calibrate our device. This allowed us to convert our resistance measurements into corresponding values of conductivity. We recorded data every 2 s. We carried out several tests to determine the optimum frequency for use in our experiments of blood conductivity. We selected the operating frequency in accordance with the results of impedance spectroscopy of blood in the broadband range from 40 Hz to 100 MHz [22, 23]. The impedance spectroscopy data show that the electrode polarization has minimal impact on frequencies above 100 kHz. Some adverse effects such as stray capacitances and inductances appear at frequencies above 5 MHz. We assessed the stability of the system using frequencies ranging from 100 kHz to 1 MHz. We found that stable measurements were obtained at a frequency of 200 kHz, and this frequency was used in all subsequent experiments. The measurements were conducted using LCR mode at voltage *V*_*ac*_ = 1 V.

We used phosphate buffered saline (PBS) and then deionized water for cleaning of the device. Before every experiment on the time-dependent changes in blood conductivity, we routinely tested our device using air, PBS, standard KCl solution, and water. The efficacy of the cleaning process was confirmed by repeating the impedance measurements on each sample.

#### Optical observation of blood sedimentation

**Fig 2:**
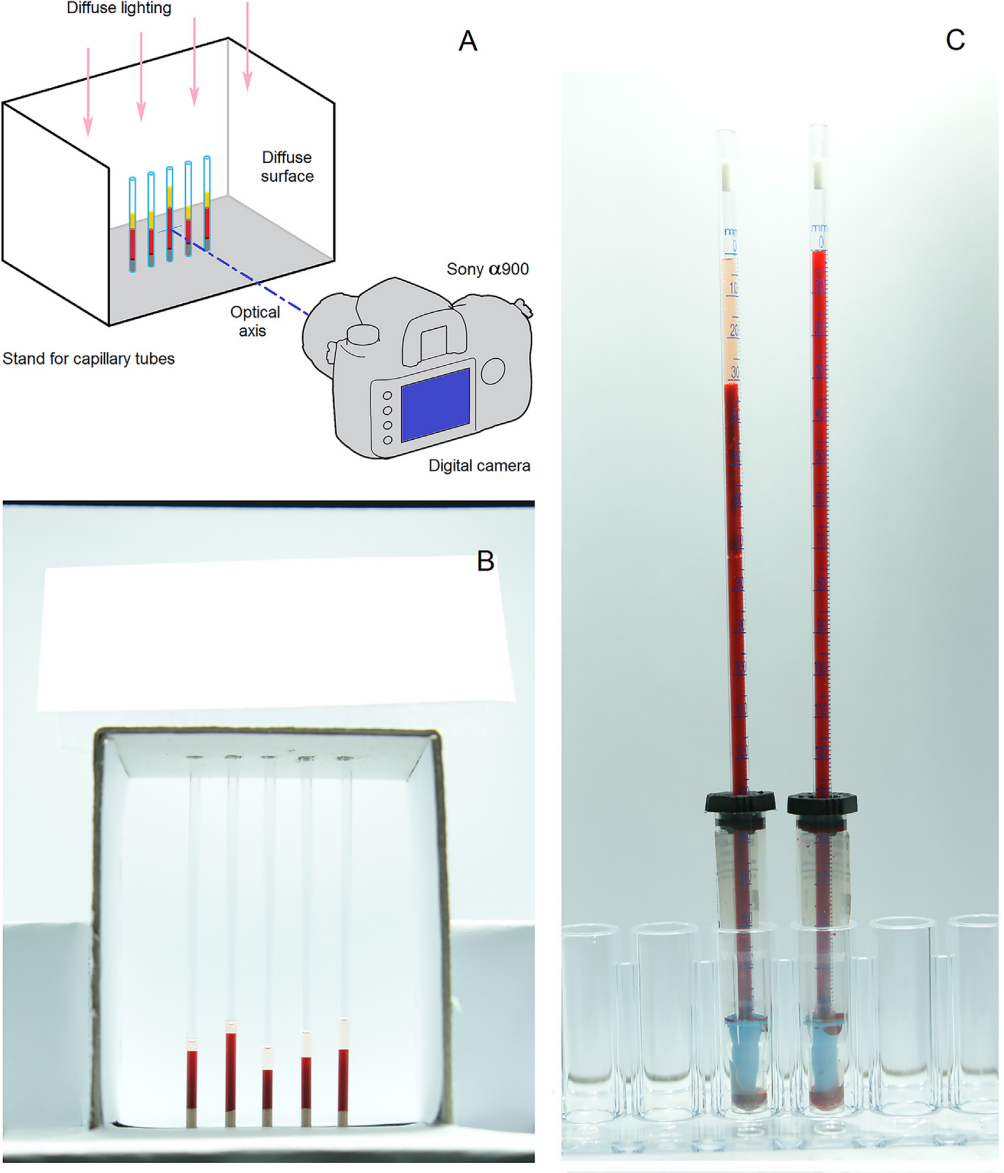
Schematic of the experimental setup that was used to obtain optical observations of blood sedimentation. (A) Sony α900 digital camera with Carl Zeiss Vario-Sonnar lens. (B) Photograph of the micro-hematocrit capillary tubes in 5-place rack. Each capillary tube has an inner diameter of 1.2 mm and a length of 75 mm. (C) Photograph of the Sedi-Rate ESR System inside the light-tight box. Each plastic tube has a sedimentation scale of 200 mm and a bore of 2.55 mm.

To measure blood sedimentation optically, we filled micro-hematocrit capillary tubes (Chase Scientific Glass, Inc., Rockwood, TN, 37854) with blood, placed them in a light-tight dark box with diffuse lighting, and took photographs at regular intervals using a digital camera (Sony α900; Sony, Corp., Tokyo, Japan). A schematic drawing of the experimental setup of the optical observations is shown in Fig 2.

#### Preparation of Blood Samples

Separated erythrocytes obtained from the Korean Red Cross were collected in a blood bag system, which contained citrate phosphate dextrose adenine 1 (CPDA-1) as the anti-coagulant. Whole blood from the bag system was centrifuged at 3000 rpm for 10 min, and the plasma and buffy coat were then removed. The resulting erythrocytes were washed three times with an isotonic phosphate buffered saline (PBS; Gibco Life Technologies, Inc., Grand Island, NY; pH = 7.4, 290 mOsmol/kg). Subsequently, the erythrocytes, plasma, and PBS were again mixed in various proportions, as required for our experiments. To investigate the effect of plasma concentration, samples were prepared with the HCT fixed at 62%, but with varying volumes of additional plasma. A range of samples was also prepared with a fixed plasma volume and different cell volumes injected into the plasma. The HCT of each sample was confirmed using a commercial HCT meter (Hanil Science Industrial Co., Ltd., Incheon, South Korea). This medical centrifuge measures the HCT using a small quantity of blood.

Dextran with a low molecular weight of 40,000 Da (D1448; Tokyo chemical industry Co., Ltd.) was used to inhibit the erythrocyte aggregation. Dextran with an average molecular weight of 100,000–200,000 Da (D4876; Sigma-Aldrich) and 425,000–575,000 Da (D1037; Sigma-Aldrich) was used to enhance the erythrocyte aggregation [24]. The dextran fractions were dissolved in plasma to get the solution with the concentration of 10 g/dl. The prepared solution were resuspended in the blood sample with HCT = 62%; the additional volume of pure plasma was added to obtain the final dextran concentration of 0.5, 1, or 1.5 g/dl and desired hematocrit level.

#### Westergren ESR test

We prepared blood samples of desired HCT and certain concentration of dextran or PBS. Next, blood samples were injected into vacuum blood collection tubes (INSEPACK, Sekisui Medical Technology (China) Ltd.), which contain ethylenediaminetetraacetic acid (EDTA-3K) as an anticoagulant. Last, we used Sedi-Rate ESR System (Globe Scientific Inc., Paramus, NJ, USA) to provide standard Westergren ESR test (see Fig 2C).

### Nomenclature and typical values of physical properties and parameters

This study covers a wide range of topics. In order to assist readers to better understand the mathematical equations and the contents, we collected notations, descriptions, units, and typical values of all used parameters in 3 Tables. Table 1 summarizes erythrocyte sizes and electrical properties of blood components required for estimating the blood conductivity.

**Table 1:** Erythrocyte sizes and electrical properties.

Notation	Description	Units	Typical value
*σ*	Blood conductivity	S/m	
*σ*_*f*_	Conductivity of plasma	S/m	1.2 at 24°C
*σ*_*m*_	Conductivity of membrane	S/m	5·10^–5^
*σ*_*cp*_	Conductivity of cytoplasm	S/m	0.5
*σ*_*px*_, *σ*_*py*_, *σ*_*pz*_	Equivalent conductivity of shell-ellipsoid		
*R*	Blood resistivity	Ohm·m	
*T*	Blood temperature	°C	
*H*	Hematocrit	per cent	
*ϕ*_*p*_	Erythrocyte volume fraction		
*R*_*ERY*_	Erythrocyte radius	μm	4
*R*	Erythrocyte equivalent radius	μm	2.78
*R*_*AGG*_	Equivalent radius of aggregated cells	Μm	
*V*_*ERY*_	Erythrocyte volume	μm^3^	90
*Δ*	Erythrocyte thickness	μm	2
*Δ*	Membrane thickness	μm	7.5·10^–3^
*a*_*x*_, *a*_*y*_, *a*_*z*_	Ellipsoid principal axis	μm	4, 4, 1
*k*	*k = x*, *y*, *z*		
*ξ*	Axis ratio		
*L*_*x*_, *L*_*y*_, *L*_*z*_	Depolarization factors		
*p*, *q*, *β*_*k*_	Auxiliary parameters		

Table 2 shows the parameters which are necessary for the study of erythrocyte sedimentation.

**Table 2:** Viscous properties of fluids, densities and velocity of sedimentation.

Notation	Description	Units	Typical value
Re	Reynolds number		
*w*	Mean velocity of erythrocyte relative to plasma	μm/s	
*w*_*t*_	Terminal velocity	μm/s	
*w*_*f*_	Velocity of plasma	μm/s	
*w*_*s*_	Settling velocity	μm/s	
*T*	Time	S	
*τ*	Particle relaxation time	S	
*μ*	Dynamic viscosity of plasma	mPa·s	1.25 at 37°C, 1.65 at 24°C
*μ*_*PBS*_	Dynamic viscosity of PBS	mPa·s	0.09 at 24°C
*ρ*_*f*_	Density of plasma	g/cm^3^	1.045
*ρ*_*PBS*_	Density of PBS	g/cm^3^	1.040
*ρ*_*p*_	Density of erythrocytes	g/cm^3^	1.095
*F*_*D*_	Drag force	N	
*C*_*D*_	Drag coefficient		
*A*	Projected frontal area of the particle		
*g*	Gravitational acceleration	m/s^2^	9.8
*f*_*ξ*_	Stokes correction factor		
*N*	Exponent in Richardson-Zaki equation		4.65

In Table 3, we have collected information related to the semi-empirical models of blood sedimentation.

**Table 3:** Notations for semi-empirical models of blood sedimentation.

Notation	Description	Units
*h*	Total height of liquid column	mm
*h*_*p*_	Height of plasma zone	mm
*h*_*b*_	Height of blood zone	mm
*h*_*∞*_	Limit to the grow of plasma zone	mm
*h*_*PBS*_	Height of PBS zone	mm
*t*_50_	Half-time of sedimentation	S
*Β*	Exponent in Puccini *et al*. semi-empirical model	
*α*	Velocity of RBC sedimentation in PBS	μm/s
*W*	Westergren rate	mm/h
*Δσ*	Difference in blood conductivity	S/m
*γ*	Exponent in equation of correlation	
*λ*	Coefficient in equation of correlation	S/m·(mm/h)^γ^
*S*	Minimal sum of squared residuals	S^2^/m^2^
$\bar{\Delta }$	Average difference	S/m
*N*	Number of experimental data	

### The morphology of erythrocytes and the electrical properties of blood

#### Morphology of the erythrocyte

We used published experimental data to provide parameters for our theoretical calculations. A review of the experimental and theoretical data relating to erythrocyte size and the electrical properties of human blood is provided in S1 Text.

**Fig 3:**
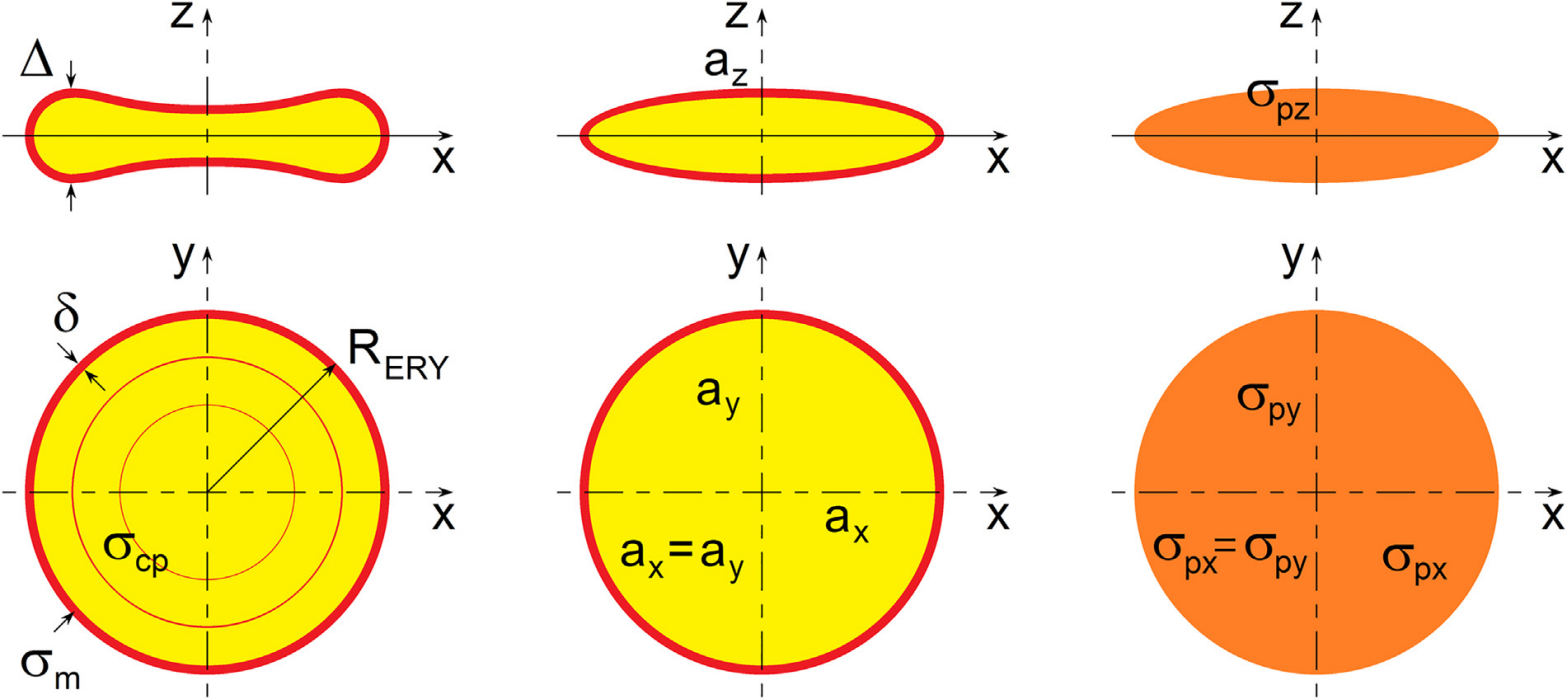
Theoretical model of the suspension of randomly oriented spheroidal particles. Shell-ellipsoid model for suspension of erythrocytes.

In the present study, we modeled erythrocytes as oblate ellipsoids encased by thin membranes (Fig 3). For our analysis of erythrocyte cell morphology, we chose the following ellipsoid dimensions to simulate the average size of erythrocytes: radius *a*_*x*_ = *a*_*y*_ = *R*_*ERY*_ = 4 μm and thickness *a*_*z*_ = *Δ* / 2 = 1 μm; membrane thickness *δ* = 7.5·10^–3^ μm. The volume of this simulated erythrocyte is *V*_*ERY*_ = 4*πa*_*x*_ *a*_*y*_ *a*_*z*_/3 = 89.4 μm^3^, which corresponds to the published experimental data. This is a suitable model because there is little difference between the electric polarizations of biconcave and oblate ellipsoid models [25].

#### Electrical resistivity of human blood

It is well established that, at a given temperature, the resistivity of blood strongly depends on its HCT [26]. Resistivity or specific resistance is the reciprocal of conductivity, *r* = 1/*σ*. In the present study, we considered both resistivity and conductivity because many experimental studies prefer resistivity, while theoretical works often rely upon conductivity. Empirically-derived relationships between blood resistivity *r* (in Ohm·cm) and hematocrit *H* (in per cent) are collected in Table 4. All the data in the table were obtained from experiments at normal body temperature (37°C).

**Table 4:** Resistivity of human blood.

Resistivity, *r* [Ohm·cm]	References
2.424 *H* + 20.916	Mohapatra and Hill (1975) [26]
54.7 exp(0.021 *H*)	Geddes and Kidder (1976) [27]
2.1022 *H* + 30.09812	Hill and Thompson (1975) [28]
49.477 exp(0.0248 *H*)	Sandberg *et al*. (1981) [29]

Mohapatra and Hill [26] also studied the dependence of blood resistivity on temperature:

r=(6.272H+75.176)−(0.104H+1.467)T, (1)

where *r* is the resistivity in Ohm·cm, *H* is the hematocrit in per cent, and *T* is the blood temperature in Celsius. Visser [30] suggested an approximate relationship between the conductivity of plasma *σ*_*f*_ and temperature *T* based on a second-order polynomial:

$$\sigma_{f}=0.335+0.0405T-0.000192T^{2},$$ (2)

where the units of *σ*_*f*_ and *T* are S/m and °C, respectively. According to this relationship, the conductivity of plasma at *T* = 24 and 37°C is *σ*_*f*_ = 1.2 and 1.57 S/m, respectively. All our experiments were performed at room temperature (i.e., 24 ± 1°C).

In our analysis, we used the following average values: conductivity of plasma *σ*_*f*_ = 1.2 S/m at a temperature *T* = 24°C, electrical conductivities of *σ*_*m*_ = 5·10^–5^ S/m for the erythrocyte cell membrane and *σ*_*cp*_ = 0.5 S/m for the erythrocyte cytoplasm.

### The method of effective medium theory

#### The effective conductivity of whole blood

We have based our method for calculating the effective conductivity of whole blood on effective medium theory. We provide a review of the use of effective medium theory to describe the electrical conductivity of dilute particle suspensions in S2 Text.

The Bruggeman effective medium theory [31] can be applied to the case of high volume fractions. In this theory, the equations for dilute suspensions is extended to high volume fraction by gradual addition of particles. An important advantage of the Bruggeman theory is its ability to describe the behavior of percolating systems, which many other approximations are incapable of describing.

We model blood as a mixture of rotational ellipsoid inclusions. We can therefore assume that *a*_*x*_ = *a*_*y*_ and consequently define the axis ratio as *ξ* = *a*_*z*_/*a*_*x*_ = *a*_*z*_/*a*_*y*_. Prolate ellipsoids (of pointy or elongated form) have *ξ* > 1, whereas oblate ellipsoids (of planetary or flattened form) have *ξ* <1. The depolarization factors (*L*) along each axis are given by [32]:

$$L_{x}=L_{y}=\{\begin{array}{cc} \frac{\xi }{4p^{3}}\left[2\xi p+\text{ln}\frac{\xi -p}{\xi +p}\right] & if\;\xi >1,\\ \frac{\xi }{4q^{3}}\left[\pi -2\xi q-2\text{arctan}\frac{\xi }{q}\right] & if\;\xi <1, \end{array}$$ (3)

$$L_{z}=\{\begin{array}{cc} \frac{1}{2p^{3}}\left[\xi \text{ln}\frac{\xi +p}{\xi -p}-2p\right] & if\;\xi >1,\\ \frac{1}{2q^{3}}\left[2q-\xi \pi +2\xi \text{arctan}\frac{\xi }{q}\right] & if\;\xi <1, \end{array}$$ (4)

in which *L_x_*+*L_y_*+*L_z_* = 1, $p=\sqrt{\xi^{2}-1}$, and $q=\sqrt{1-\xi^{2}}$.

The thickness of the erythrocyte membrane *δ* is very small compared with the erythrocyte radius, *R*_*ERY*_, and its thickness, *Δ* (see Fig 3). Hence, *δ/a*_*x*_, *δ/a*_*y*_, and *δ/a*_*z*_ << 1, and the volume ratio *η* of the inner ellipsoid to the outer ellipsoid may be approximated by [32]:

$$\eta \approx \left(1-\frac{\delta }{a_{x}}\right)\left(1-\frac{\delta }{a_{y}}\right)\left(1-\frac{\delta }{a_{z}}\right).$$ (5)

The equivalent conductivity of a shell-ellipsoid is a tensor that has three components along the *x*-, *y*- and *z*-axis of the ellipsoid: *σ*_*px*_, *σ*_py_, and *σ*_*pz*_, respectively. For an axis *k* (*k* = *x*, *y*, *z*), the component *σ*_*pk*_ can be expressed as [32]:

$$\sigma_{pk}=\sigma_{m}\frac{\beta_{k}\sigma_{m}+\sigma_{cp}-\beta_{k}\eta (\sigma_{m}-\sigma_{cp})}{\beta_{k}\sigma_{m}+\sigma_{cp}+\eta (\sigma_{m}-\sigma_{cp})},$$ (6)

where *σ*_*m*_ and *σ*_*cp*_ are the electrical conductivity of the membrane and cell cytoplasm, respectively, and *β*_*k*_ = (1-*L*_*k*_)/*L*_*k*_. In this work, we assume that the erythrocytes have random orientations and are randomly distributed in the plasma. Thus, the effective conductivity of the blood is a scalar, despite the fact that the conductivity of each cell is a tensor.

We used the approaches proposed by Asami [32] and Giordano [33] to derive our relationship for the effective conductivity of erythrocytes where the particle volume fraction *ϕ*_*p*_ < 0.1:

$$\sigma =\sigma_{f}+\frac{\frac{1}{3}\varphi_{p}\sigma_{f}\sum_{k}^{x,y,z}\frac{\left(\sigma_{pk}-\sigma_{f}\right)}{\sigma_{f}+L_{k}(\sigma_{pk}-\sigma_{f})}}{1+\varphi_{p}\left[\frac{1}{3}\sigma_{f}\sum_{k}^{x,y,z}\frac{1}{\sigma_{f}+L_{k}(\sigma_{pk}-\sigma_{f})}-1\right]},$$ (7)

where *σ*_*f*_ is the conductivity of plasma. This last equation is similar to an equation proposed by Giordano *et al*. [34] if the principal conductivities are defined by Eq 6. The application of the so-called Bruggeman or differential technique to the case of large volume fractions is thoroughly described in the work of Giordano *et al*. [34].

It is simple to extend the equations for an ellipsoid covered by a single thin membrane to more complicated multilayered systems. However, the dielectric properties of the different membrane layers are not yet totally known, although several studies have been reported [35–37].

Unfortunately, the current theory does not allow us to fully characterize the dense suspension of erythrocytes in plasma. The application of numerical methods for the analysis of such systems is very complicated. Therefore, we approximated the shape of the red blood cell by an ellipsoid and used the existing effective medium theory to analyze the experimental data.

#### Comparison of theoretical and experimental data for the effective conductivity

**Fig 4:**
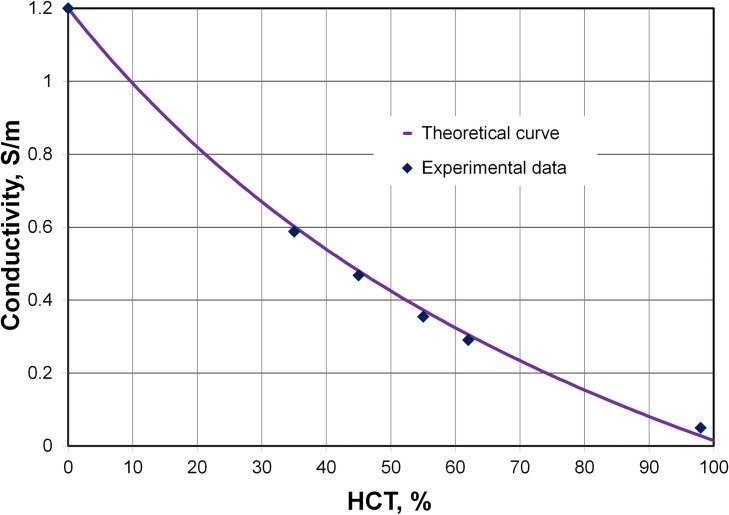
Comparison of the effective medium theory and the experimental data. Conductivities at various hematocrits (HCTs) were calculated using Eq 7. Our chamber with two planar electrodes was used to measure conductivity. Five samples of washed erythrocyte suspensions in pure plasma were prepared at HCT = 35, 45, 55, 62, and 98%.

Fig 4 presents our experimental and theoretical result for the change in blood conductivity with HCT. The theoretical results of other authors are shown in S2 Text for comparison. Based on published data, we used the same set of physical parameters to plot all of the theoretical curves including our curve. We assumed a mixture of disaggregated erythrocytes in the shape of oblate spheroids with *a*_*x*_ = *a*_*y*_ = *R*_*ERY*_ and *a*_*z*_ = *Δ* / 2. Using Eqs 3 and 4, we calculated the depolarization factors *L*_*x*_ = *L*_*y*_ = 0.1482 and *L*_*z*_ = 0.7036. The principal conductivities given by Eq 6 were *σ*_*px*_ = *σ*_*py*_ = 0.0281 S/m and *σ*_*pz*_ = 0.00623 S/m.

Obviously, the conductivities calculated using theoretical method [solid violet line, based on Eq (7)] show good agreement with our experimental data in Fig 4.

An analysis of blood resistivity for a range of HCT values from published experimental studies (Table 4) was presented in our previous work [20]. We concluded that, as compared with the other equations, our Eq 7 provides better agreement with the experimental data that we collected on the conductivity of the disaggregated erythrocyte suspension, and is in good agreement with other experimental data on the resistivity of blood.

### The fall of a single particle in a viscous fluid: Basic equations

The Reynolds number, Re, provides a measure of the ratio of inertial to viscous forces. The Reynolds number for a spherical particle is [38]:

$$\text{Re}=\frac{2\rho_{f}wR}{\mu },$$ (8)

where *ρ*_*f*_, is the density of the fluid, *w* is the mean velocity of the sphere relative to the fluid, *R* is the sphere radius, and *μ* is the dynamic viscosity of the fluid. The drag coefficient *C*_*D*_ is the ratio of the drag force divided by the projection area to the dynamic pressure [38]:

$$C_{D}=\frac{F_{D}/A}{\rho_{f}w^{2}/2},$$ (9)

where *F*_*D*_ is the drag force and *A* is the projected frontal area of the particle (for a sphere *A* = *πR* ^2^). For low Reynolds numbers Re << 1, it has been shown by experiment that the drag coefficient obeys the equation:

$$C_{D}=\frac{24}{\text{Re}}.$$ (10)

Stokes’ law follows from Eqs 8 and 9:

$$F_{D}=6\pi \mu Rw.$$ (11)

The equation of motion of a single sphere in a viscous fluid is:

$$\frac{4}{3}\pi R^{3}(\rho_{p}-\rho_{f})\frac{dw}{dt}=\frac{4}{3}\pi R^{3}(\rho_{p}-\rho_{f})g-F_{D},$$ (12)

where *ρ*_*p*_ is the density of particle and *g* is the acceleration due to gravity.

If the drag force is balanced by the buoyancy force and the effect of gravity, then the resulting settling velocity (or terminal velocity *w*_*t*_) is given by:

$$w_{t}=\frac{2(\rho_{p}-\rho_{f})R^{2}g}{9\mu }.$$ (13)

Eq 13 indicates that particles with greater mass will also have higher velocity. The *particle relaxation time* is the characteristic time for a particle to make the transition from one state to another, or from rest to motion with constant velocity in this case. The relaxation time of a spherical particle *τ* is given by:

$$\tau =\frac{2(\rho_{p}-\rho_{f})R^{2}}{9\mu }.$$ (14)

## Results and Discussion

### Time dependence of blood conductivity

**Fig 5:**
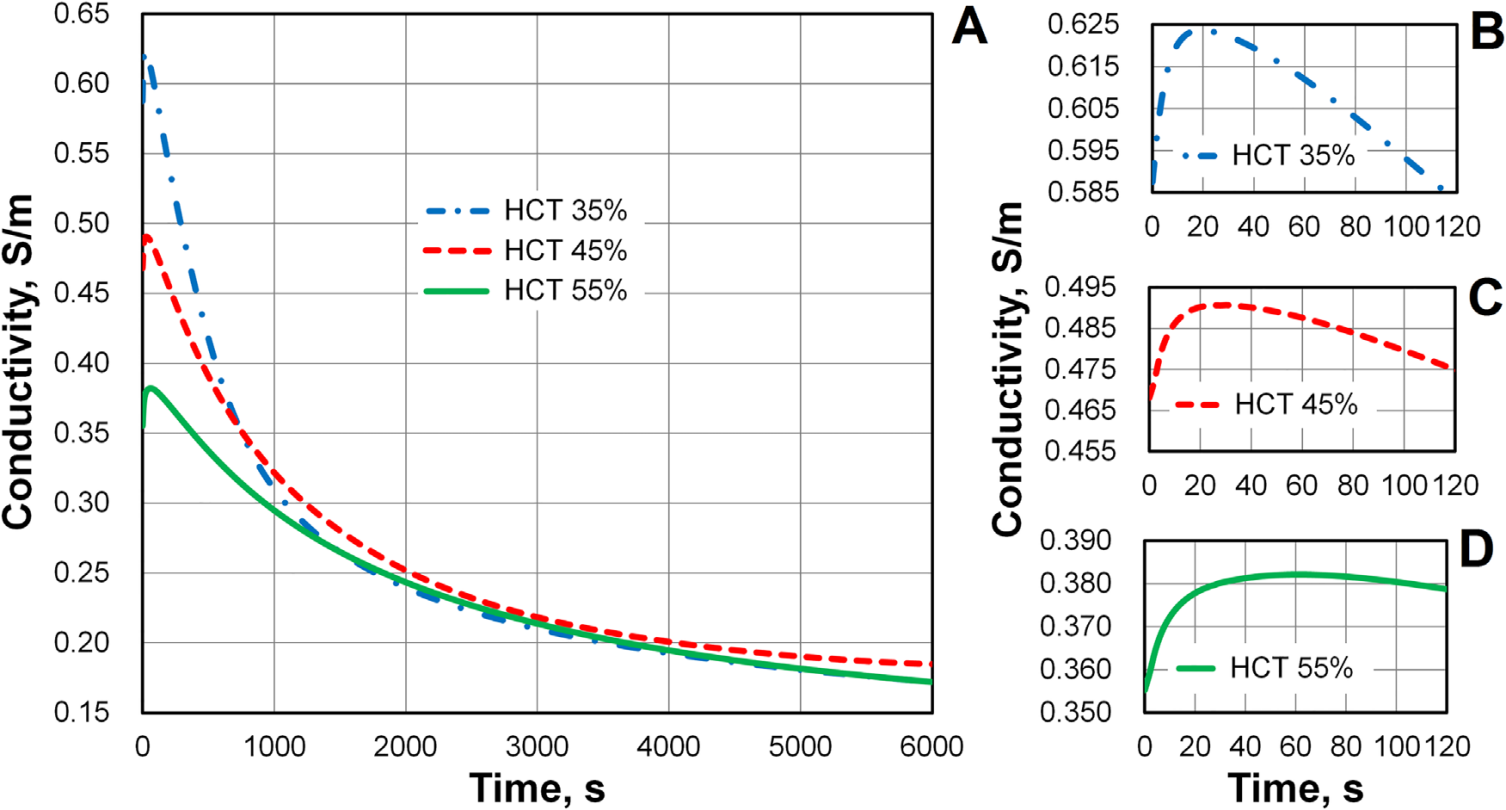
Time-dependent changes in blood conductivity during sedimentation. Three samples of washed erythrocyte suspensions in pure plasma were prepared at hematocrit (HCT) = 35, 45, and 55%. (A) The conductivity of blood increased slightly during the first minute of observation and then decreased for more than 1.5 h. The conductivities of the disaggregated erythrocyte suspension were *σ* = 0.588, 0.468, and 0.355 S/m, at HCT = 35, 45, and 55%, respectively. The conductivity of the deposit on the bottom of the chamber was approximately 0.175 S/m at the end of the sedimentation, regardless of the initial HCT. (B) Initial changes in blood conductivity at HCT = 35%. The maximum value of the initial increase in conductivity was about 0.036 S/m at 18s. (C) The maximum value of the initial increase in conductivity was 0.028 S/m at 31 s (HCT = 45%). (D) The maximum value of the initial increase in conductivity was 0.027 S/m at 60 s (HCT = 55%).

We measured the conductivity *σ* of whole blood samples with HCT values of 35, 45, and 55% over a period of 6000 s. The representative behavior of changes in conductivity over time are shown in Fig 5. We used four different blood bags to prepare the necessary samples separately. Each measurement was repeated three times. The curves display the average values over these measurements. The standard deviation in the conductivity was less than 0.005 S/m at each time point of measurement. These curves are very close to the curves obtained in the chamber of round cross-section [20]. We assume this shows that our method is very reliable. It is practically independent on the shape of chamber and the electrode configuration. Thus, the time course of conductivity reflects the actual behavior of blood.

Conductivity only has a physical meaning for true solutions. Blood is a homogeneous suspension of mixed particles, so we have technically measured *effective* conductivity, rather than conductivity itself. A column of blood was held in a chamber during the experiments (Fig 1A). During sedimentation, the conductivity of the blood varied depending on the height in the column; our reported values are averages. Initially, the conductivity corresponded to the conductivity of a homogeneous blood suspension. However, at the end of sedimentation, the conductivity was very close to that of the deposit.

Fig 5A shows that the conductivity of the blood in the chamber increased during the first minute of observation. The conductivity subsequently began to decrease, and continued to do so for more than 1.5 h. The period of increasing conductivity is shown in greater detail in Fig 5B–5D. An initial increase in conductivity with time was also observed by Pribush *et al*. [12]. This effect is small, but the accuracy of our method allowed it to be noticed.

We connected the impedance analyzer to the empty chamber, injected the erythrocyte suspension into the chamber using a pipette, and continued to record the conductivity. In our experiments, the increase in the conductivity took roughly 1 s to begin. We considered the beginning of the increase in conductivity as the starting point of our measurements. The conductivities of the disaggregated erythrocyte suspensions with HCT = 35, 45, and 55% were *σ* = 0.588, 0.468, and 0.355 S/m, respectively. We attributed the increase in the blood conductivity that occurred during the first minute to erythrocyte aggregation. Subsequently, the conductivity decreased as the erythrocytes deposited at the bottom of the chamber.

The maximum values of the initial increases in conductivity were about 0.036 S/m at 18 s, 0.028 S/m at 31 s, and 0.027 S/m at 60 s with HCT = 35, 45, and 55%, respectively. We noticed that the conductivity took a longer time to reach its maximum value as the HCT increased. We assumed that this was explained by the formation of longer aggregates that accompanies higher HCTs. Further, the subsequent, decreasing behavior of the conductivity was assumed to reflect erythrocyte sedimentation. Higher HCTs reduce the sedimentation rate.

**Fig 6:**
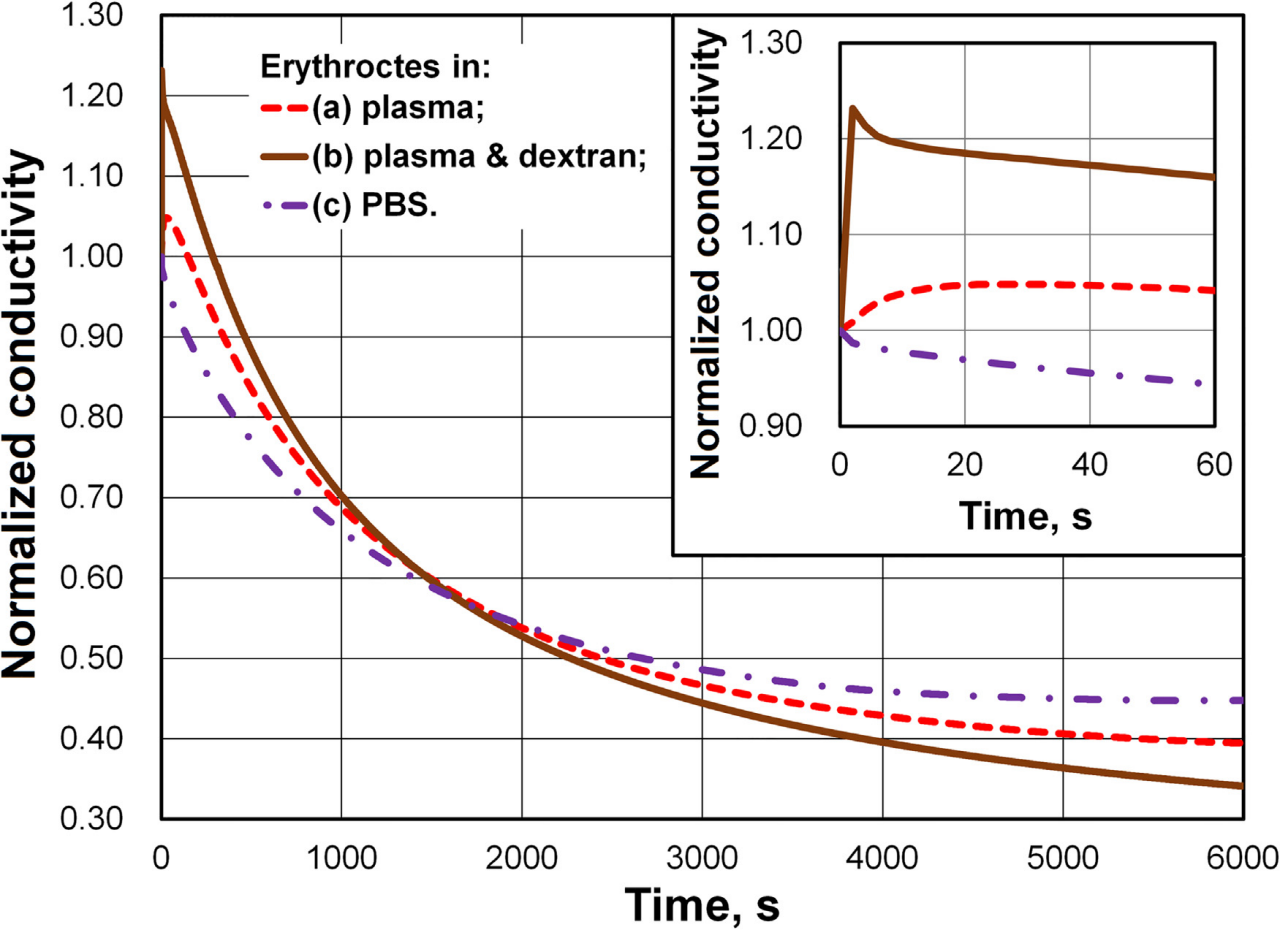
Influence of dextran and phosphate buffered saline (PBS) on changes in blood conductivity during sedimentation. The plot presents a comparison of the changes in conductivity for three suspension of erythrocytes in (a) pure plasma, (b) PBS (pH = 7.4, 290 mOsmol/kg), and (c) dextran (425~575 kDa, 1 g/dl) solution in plasma. Each sample had a hematocrit = 45%. The inset presents the details of the initial stage of the change in conductivity.

To test our assumptions, we prevented red blood cell aggregation from having an effect on the time course of conductivity by suspending the cells in PBS (Fig 6). Additionally, we used dextran from *Leuconostoc mesenteroides* with an average molecular weight of 425,000–575,000 Da to enhance aggregation, as it is known that the dextran of high molecular weight increases the rouleaux formation [24]. The concentration of dextran was 1 g/dl. For comparison with Figs 5 and 6 presents the time course of the conductivity of the erythrocyte suspension in plasma with HCT = 45%. Each of the samples in Fig 6 had HCT = 45%. All curves in Fig 6 were normalized using the initial conductivity (at time *t* = 0) of corresponding blood sample. The initial conductivities of the disaggregated erythrocyte suspensions in plasma, plasma with dextran, and PBS were *σ* = 0.468, 0.369, and 0.521 S/m, respectively. The conductivities of pure plasma, plasma with dextran, and PBS were *σ*_*f*_ = 1.2, 0.904, and 1.452 S/m, respectively. Fig 6 shows that dextran of a high molecular weight (425,000–575,000 Da) enhanced aggregation. In this case, the increase in blood conductivity took less than 2 s. The conductivity of the dextran sample then decreased more rapidly than had the conductivity of erythrocytes in plasma. In contrast, the conductivity of the erythrocyte suspension in PBS showed no growth and decreased more slowly than the conductivity of erythrocytes in plasma. Here, changes in blood conductivity during sedimentation also depended on the viscosity and conductivity of the liquid.

Change in the blood conductivity may also result from realignment of cells in the liquid due to the electrophoretic or dielectrophoretic forces in strong electric field. Moreover, a non-uniform electric field could crenate and shrink erythrocytes, dynamically change plasma conditions, and increase the blood conductivity, as discussed by An *et al*. [39]. However, we estimate that these effects are negligible in our case, because the electric field is too weak. The dynamic influences of dextran on the rheological and electrical properties of erythrocyte suspensions and changes in the erythrocyte morphology were studied by Antonova *et al*. [40, 41]. Nevertheless, we suppose that in our case this dynamic effect is also negligible, since the sedimentation rate is insignificant in comparison with the fluid velocity in a Couette rheometer, where the dynamic effect is noticeable.

One of our goals was to develop and test an easy-to-use device that would be suitable for potential application in point-of-care diagnostics. Therefore, we decided not to use an additional system that would ensure the initial disaggregation of the erythrocytes. Before starting the measurements, we used a vortex mixer for several seconds to disaggregate the blood samples. Next, we gently inverted the samples a minimum of 10 times to obtain a completely homogeneous suspensions. In addition, we assumed that the red blood cells would be subject to enough stress to result in disaggregation during movement through the pipette. For a detailed study of the initial stage of aggregation, we plan to use mechanical vibration as, for example, has been used by Pribush *et al*. [12, 17]. Apparently, the use of mechanical vibration also requires more detailed study, because it is difficult to agitate the fluid inside the small chamber and control the homogeneity of volume fraction distribution.

Pribush *et al*. [12, 17–19] observed the complex kinetics of erythrocyte aggregation in their measuring chambers, in which the electrodes were placed at the upper region of the blood column. The electrode geometry in our chamber does not allow us to track the change in conductivity with precision in the upper portion of the blood column. The measured time dependence of the blood conductivity is a very smooth function (see Fig 5A). It does not show even a weak reflection of the collapse and fragmentation of the erythrocyte network formation, as discussed by Pribush *et al*. [12, 17–19].

### The influence of erythrocyte aggregation on the effective conductivity of blood

Erythrocyte aggregation affects whole blood conductivity. Erythrocytes can form rouleaux aggregates, which resemble a stack of coins.

In our previous work [20], we calculated the change in blood conductivity with aggregate size. The increase in conductivity was approximately 0.075 S/m for all levels of HCT, as compared with the conductivities of the corresponding disaggregated suspensions. In our experiments, we observed a maximum conductivity increase of 0.02 S/m (Fig 5B–5D). In reality, the experiments included a mixture of erythrocytes with various aggregate sizes at different times after the start of measurement. Therefore, the experimental increase in conductivity should not be expected to equal the maximum achieved in the theoretical calculations. Aggregation and sedimentation are strongly coupled. Aggregation increases both the blood conductivity and the sedimentation rate, as will be discussed below. In contrast, sedimentation reduces the conductivity measured at the bottom of the chamber. It was only possible for us to provide an approximate evaluation of the increase in conductivity that accompanied the growth of aggregates. However, theoretical calculations show that the conductivity quickly becomes saturated as the aggregate sizes increase [20]. Nevertheless, aggregation continues to affect conductivity about 60 s after the beginning of sedimentation because the conductivity of the disaggregated erythrocyte suspension is smaller than the conductivity of the aggregated blood. Apparently, the initial stage of linear aggregate formation transforms into a phase that is characterized by network shaping [12]. However, we assume that this transformation has an insignificant influence on the effective conductivity of blood.

### Estimation of the settling velocity and particle relaxation time

The apparent viscosity of blood depends on the shear rate and exhibits the non-Newtonian behavior. At a normal physiological HCT of 45% and at a shear rate of 1000 s^-1^ or higher, the dynamic viscosity of blood is approximately 4 mPa s, which is roughly 4 times that of water. Plasma (zero HCT) has a viscosity *μ* = 1.1–1.6 mPa s, depending on the concentration of plasma proteins [42]. The dynamic viscosity of plasma is also strongly dependent on temperature. It varies from *μ* = 3.1 mPa s at 4–5°C to *μ* = 1.25 mPa at 35–37°C [43, 44]. Plasma is a Newtonian liquid, its viscosity is independent of shear rate [45]. The density of an erythrocyte is *ρ*_*p*_ = 1.089–1.100 g cm^-3^, according to Sethu *et al*. [46], or *ρ*_*p*_ = 1.093–1.097 g cm^-3^, according to Kenner [47]. The density of blood plasma at 37°C is *ρ*_*f*_ = 1.046–1.049 g cm^-3^ [47].

For the remainder of this analysis, we chose the following average values of the parameters: the density of plasma *ρ*_*f*_ = 1.045 g cm^-3^; the density of erythrocytes *ρ*_*p*_ = 1.095 g cm^-3^; the dynamic viscosity of plasma *μ* = 1.25 mPa s at 37°C; and the mean volume of erythrocyte *V*_*ERY*_ = 90 μm^3^. A sphere with an equivalent volume to an erythrocyte has a radius *R* = 2.78 μm. The Reynolds number is extremely low, Re < 10^–5^. Using these parameters, we estimated the terminal velocity of individual insulated erythrocyte to be *w*_*t*_ = 0.673 μm/s = 2.42 mm/h, and the relaxation time of a single erythrocyte to be *τ*_*ERY*_ = 1.61 μs. This relaxation time was very short compared with the time taken for sedimentation. Thus, in a suspension of non-interacting particles, each particle would reach terminal velocity about 2 μs after the beginning of deposition.

### Corrections for the drag force due to high hematocrit and the non-spherical shape of erythrocytes

When erythrocytes settle at a high HCT (about 30–50%), they provoke an opposite, upwards flow of the plasma. Suppose that all erythrocytes have the same velocity *w*, then to ensure the conservation of volume:

$$w\varphi_{p}=-w_{f}(1-\varphi_{p}),$$ (15)

where *w*_*f*_ is the velocity of plasma. For a suspension of non-interacting spheroidal particles the correction of Stokes’ law is [48]:

$$F_{D}=6\pi \mu R(w-w_{f})f_{\xi },$$ (16)

where *f*_*ξ*_ denotes the Stokes correction factor for spheroids. For the ellipsoid shape of particles defined in Fig 3, the aspect ratio is given by *ξ* = *a*_*z*_/*a*_*x*_. The Stokes correction factors for spheroids [48] are:

$$f_{\xi z}=\left(\frac{4}{5}+\frac{\xi }{5}\right)\xi^{-1/3}\;\text{and}\;f_{\xi x}=\left(\frac{3}{5}+\frac{2\xi }{5}\right)\xi^{-1/3}.$$ (17)

Here, *f*_*ξ z*_ is the Stokes correction factor if the erythrocyte moves along its z-axis and *f*_*ξ x*_ is the factor for motion along the x-axis. Assuming our erythrocyte model has an aspect ratio *ξ* = 0.25, we used Eq 33 to calculate that *f*_*ξ x*_ ≈ 1.111 and *f*_*ξ z*_ ≈ 1.349.

Using Eqs 13, 15 and 16 the terminal velocity of erythrocytes at high HCT is

$$w_{t}=\frac{V_{ERY}(\rho_{p}-\rho_{f})(1-\varphi_{p})g}{6\pi \mu Rf_{E}},$$ (18)

where *V*_*ERY*_ = 4*πR* ^3^/3 is the volume of erythrocyte. In Eq 18, we considered a dilute suspension of non-interacting particles. In a large number of experiments, it has been demonstrated that a dense cloud of particles settling in a fluid has a terminal velocity lower than is predicted by Stokes’ law [49]. Richardson and Zaki [50] proposed a semi-empirical correction factor for Stokes' law that incorporated the following dependence:

$$\frac{w_{s}}{w_{t}}={\left(1-\varphi_{p}\right)}^{n},$$ (19)

where *w*_*s*_ is the settling velocity at a high volumetric concentration of particles and *n* is an empirically determined exponent. Richardson and Zaki [50] showed that *n* is a function of the Reynolds number alone (in the absence of wall effects). The empirical value for the exponent *n* for low Reynolds number (Re < 0.2) is *n* = 4.65, and this is the value that we used in the present study. This value of the exponent *n* has been confirmed in many studies [49, 51]. For example, according to Eq 19, the settling velocity for HCT = 55% is 40 times smaller than the terminal velocity of a solitary particle. Research on the sedimentation of dense suspensions has more than a century’s worth of history. For a detailed analysis of the problem, we refer the reader to reviews by Yang and Renken [51] and Di Felice [49], as well as to the references therein.

We calculated the settling velocities of erythrocytes during sedimentation at different HCT values using Eqs 18 and 19 (Table 5). Here, we assume that the dynamic viscosity of plasma is *μ* = 1.65 mPa s at room temperature (24°C), as follows from an interpolation of the available experimental data [43, 44].

**Table 5:** Calculated erythrocyte settling velocities at different hematocrits.

Hematocrit	*w*_*sz*_, μm/s	*w*_*sx*_, μm/s
~0% (single erythrocyte)	0.377	0.459
35%	0.0545	0.0661
45%	0.0256	0.0312
55%	0.0104	0.0126

Note that a dense cloud of disaggregated particles settling in a fluid has a terminal velocity that is ten times smaller than the terminal velocity of a solitary particle in quiescent, unbounded liquid. This follows from the Richardson-Zaki law [50]. The erythrocytes would move at a constant settling velocity if there was no aggregation. As a result of aggregation, the weight of the agglomerates increases and the sedimentation rate is also increased, following Eq 13. Thus, the motion is accelerated.

**Fig 7:**
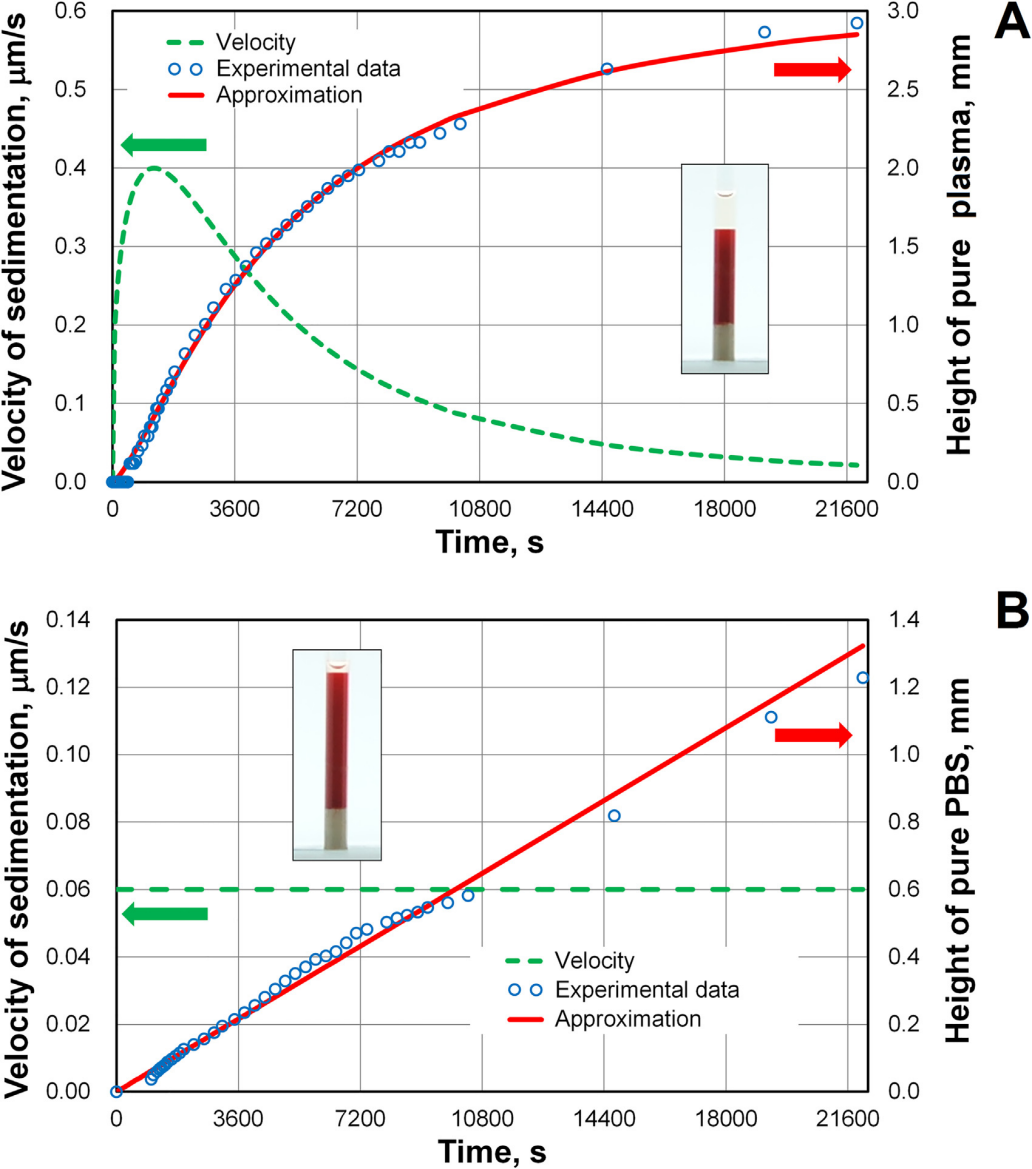
Sedimentation curve and change in the velocity of sedimentation with time for whole blood and a suspension of erythrocytes in phosphate buffered saline (PBS). (A) Sigmoid sedimentation curve and change in the sedimentation velocity of whole blood. Suspension of washed erythrocytes in pure plasma, hematocrit (HCT) = 35%. (B) Linear growth of the pure PBS zone for the suspension of erythrocytes in PBS (pH = 7.4, 290 mOsmol/kg), HCT = 45%. The insets show capillary tubes with blood after 9190 s of sedimentation.

To test the theoretical approaches, we filled capillary tubes with blood and used a digital camera to photograph the sedimentation. The inner diameter of each capillary was 1.2 mm. Experimental data on the suspension of erythrocytes with plasma at an HCT = 35% are shown Fig 7A. In the standard Westergren test, the ESR may be described by a sigmoid shaped curve [7]. We demonstrated the presence of this shape for small thin tubes. The height of blood column was 8.13 mm. Also shown in Fig 7A is a fit of the semi-empirical model proposed by Puccini *et al*. [6] to the data:

$$h_{p}(t)=h_{\infty }\left[1-\frac{1}{{\left(t/t_{50}\right)}^{\beta }+1}\right],$$ (20)

where *h*_*p*_ is the height of the plasma zone, *h*_*∞*_ is the limit to the growth of the plasma zone, *t*_50_ is the half-time of sedimentation, *β* is the exponent, and *t* is time of sedimentation. The obtained fitting parameters were *h*_*∞*_ = 3.252 mm, *t*_50_ = 5084 s, and *β* = 1.344. The velocity of sedimentation was calculated by taking the time-derivative of Eq 20:

$$w_{p}(t)=h_{\infty }\beta t_{50}^{\beta }\frac{t^{\beta -1}}{{\left(t^{\beta }+t_{50}^{\beta }\right)}^{2}}.$$ (21)

We found that the maximum velocity of sedimentation was *w*_*max*_ = 0.4 μm/s at *t* = 1228 s (Fig 7A), which is higher than the settling velocities of disaggregated cells at HCT = 35%, *w*_*sz*_ = 0.0545 and *w*_*sx*_ = 0.0661 μm/s (Table 5). Using the ratio *w*_*max*_/*w*_*sx*_ = 7.3 = *R*_*AGG*_^2^/*R* ^2^ or the ratio *w*_*max*_/*w*_*sx*_ = 6.1 = *R*_*AGG*_^2^/*R* ^2^ (where *R*_*AGG*_ is the equivalent radius of aggregated cells) we were able to determine that *R*_*AGG*_^3^/*R* ^3^ ≈ 15. The inset in Fig 7A shows a clear boundary between the plasma and blood. This indicates that the smallest aggregate was composed of about 15 cells at *t* = 1228 s near the boundary between pure plasma zone and blood column. The sedimentation curve becomes sigmoid because the erythrocyte suspension in plasma is accompanied by aggregation [52]. We assume that the aggregate size continues to grow further (after 1228 s for this sample), but the velocity of boundary between plasma and blood is reduced due to increase in the erythrocyte volume fraction. Unfortunately, the direct observation and measurement of the aggregate size is difficult.

In contrast, the sedimentation curve for the suspension of erythrocytes in PBS demonstrates linear behavior (see Fig 7B):

$$h_{PBS}(t)=\alpha t,$$ (22)

where *h*_*PBS*_ is the height of the pure PBS zone and *α* is the slope of the sedimentation curve (i.e., the velocity of sedimentation). The dynamic viscosity of PBS is *μ*_*PBS*_ = 0.90 mPa s at a temperature of 24°C. For the suspension of erythrocytes in PBS with HCT = 45%, we experimentally found that *α* = 0.06 μm/s. This value is in a good agreement with the theoretical value *α* = 0.057 μm/s that was obtained from Eqs 18 and 19 for the disaggregated cell suspension. Linear behavior Eq 22 was observed during the entirety of the experiment occurring after 20000 s.

### The relationship between the ESR and blood conductivity

**Fig 8:**
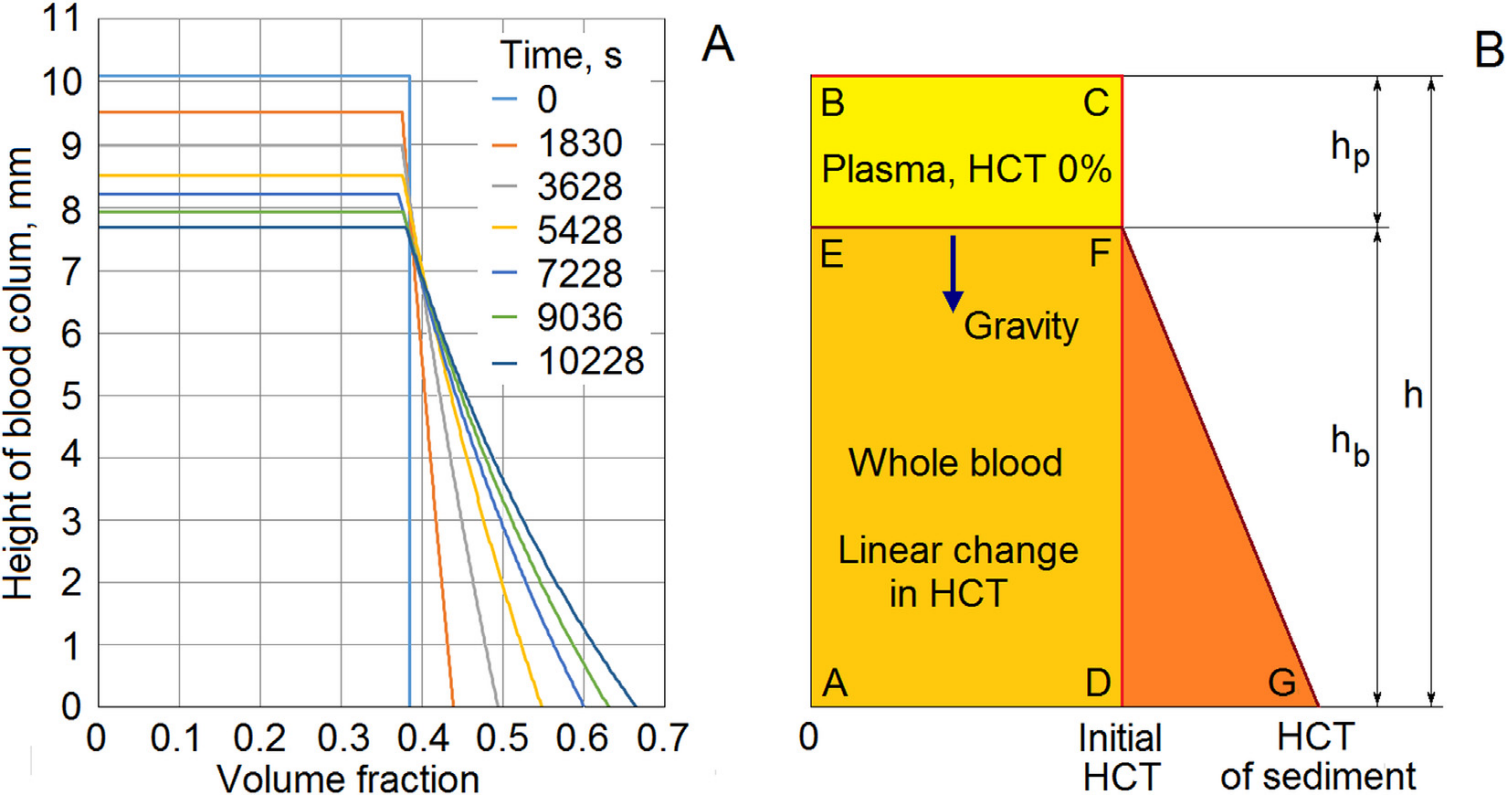
Volume fraction profile and a simple model of erythrocyte sedimentation. The height of the liquid column *h* remains constant in the process of sedimentation. The height of the plasma zone *h*_*p*_ and the volume fraction at the bottom of the blood column both increase. The velocity of the boundary between the zones changes with time. (A) Experimental observation of variation of volume fraction profile during sedimentation. Suspension of washed erythrocytes in pure plasma, initial hematocrit = 38.5%. (B) Simplified model of erythrocyte sedimentation.

In Fig 8, we present a volume fraction profile (Fig 8A) and a simple model of a column of blood during sedimentation (Fig 8B). The volume fraction profile was found by analyzing pictures taken with a digital camera. In this experiment, we again used the capillary tubes with 1.2-mm inner diameters.

In Fig 8A, the initial volume fraction of red cells in plasma was 0.385 (or HCT = 38.5%). The height of the blood column was 10.06 mm. Profiles of the volume fraction are shown at 0, 1830, 3628, 5428, 7228, 9036, and 10228 s of sedimentation. Analyzing the volume fraction distribution led us to accept that, at first approximation, the volume fraction of red blood cells remained constant on the boundary between the plasma zone and the blood zone (Fig 8B). Subsequently, the volume fraction rose monotonically to the bottom of the blood column. As a first approximation, we assume that the volume fraction changed linearly. The total volume of the red cells was maintained. Thus, the area of rectangle *ABCD* equals the area of trapezoid *AEFG* (Fig 8B).

In contrast, a different density profile for the deposition of disaggregated particles was obtained theoretically by Thelen and Ramirez [53] and Nguyen and Ladd [54]. They found that the density profile is composed of an upper portion of the fluid, a bulk region in which the concentration is constant, and a deposit at the bottom of the column. In previous work [20], we used this theoretical profile. Our new experimental data show that this is not entirely true. We assume that the difference between theoretical shape of density profile and our observations results from the aggregation of red blood cells.

Our experimental method of measuring conductivity is very sensitive to the beginning of sedimentation, and generally provides a correct reflection of the process of deposition. We have shown that blood conductivity only increases slightly during aggregation (Fig 5A). We can therefore assume that a constant electrical conductivity is maintained in the top portion of the blood zone during deposition. It is possible to estimate the value of conductivity in the top portion of the blood based on the initial stage of the time course of conductivity during sedimentation (Fig 5). The height of the plasma zone, *h*_*p*_, increased over time, but the thickness of the blood zone, *h*_*b*_, decreased over time (Fig 8). In our measurement chamber, the distance between the electrodes is very small. Accordingly, it allowed us to measure the conductivity of the deposit near the bottom of the blood column. In the case of a normal human blood with average physiological parameters of the plasma and erythrocytes, Eq 7 makes it easy to convert from the conductivity of the blood to the volume fraction of erythrocytes (the hematocrit). The graphical plot in Fig 4 may also be used for this purpose. Thus, the hematocrit can be determined on both the top and bottom of the blood column. Using the equality of the areas of rectangle *ABCD* and trapezoid *AEFG* (Fig 8B), we were able to identify the height of the plasma zone, *h*_*p*_. As a result, we were able to determine the relationship between the ESR and blood conductivity.

Our sedimentation model (Fig 8) is relatively simple. Even so, our theoretical estimates of settling velocity are similar to the rate of erythrocyte sedimentation that was evaluated from experimental data. In future investigations, it would be interesting to develop our model to incorporate the phenomena relevant to blood conductivity that have been observed in experimental work. For example, the experimental results of several researchers have shown that, in blood columns, conductivity varies with sedimentation in a complicated manner [12, 17]. We could therefore consider incorporating a more complex model of the sedimentation process. Pribush *et al*. [12] have discussed an interesting mechanism of erythrocyte sedimentation that is connected with network formation, network fragmentation, and channeling in settling blood. Unfortunately, we found no evidence either for or against this mechanism in our experiments. Perhaps the blood column was not tall enough to allow a network to be formed in our experiments. However, it is well documented that the network formation takes place for aggregative samples at static or very low shear conditions and at normal or elevated hematocrits [12]. In our case, we can assume that the network intensively forms at the bottom of the chamber, since the bottom conditions are almost static, and, in addition, the hematocrit increases with time to more than 60% (Fig 8A). Presumably, the network is formed and stays on the surface for a long time, as the shear forces required to break it are not strong enough. However, even if network collapses, we can only measure a monotonic decrease in blood conductivity at the bottom of the chamber. In our future research, we hope to investigate the network formation by means of the broadband dielectric spectroscopy. Here, we have presented a useful model of sedimentation that may be adapted as the understanding of the sedimentation process develops.

### The correlation between the ESR test and the changes in blood conductivity

In the previous subsection we proposed an algorithm for the reconstruction of the sedimentation curve using the recorded changes in the blood conductivity. This discussion was related to a small chamber. The height of our PDMS chamber is 5 mm; the normal sedimentation rate of the Westergren test is about of 10–20 mm/h. The experimental data show that the sedimentation rate in the short tube is slower than the rate in a standard tube that includes a 200 mm blood column. We explain this by the fact that the blood deposit rises from the bottom of approximately the same velocity in both cases. Consequently, the deposit reaches the boundary between pure plasma and blood in the short tube in a shorter time. When the deposit is almost reached the boundary, the erythrocyte volume fraction increases near the boundary between the pure plasma and blood, so that it slows down the settling velocity. This leads to a low sedimentation rate in short tubes. However, we assume that the first 400–600 s of conductivity recording correctly reflected the behavior of the blood not only in our small chamber, but also the behavior of the same blood sample in standard Westergren tube. In this subsection, we compare our method with the standard Westergren test by investigating the same blood samples with both methods to inquire the correlation between them.

**Fig 9:**
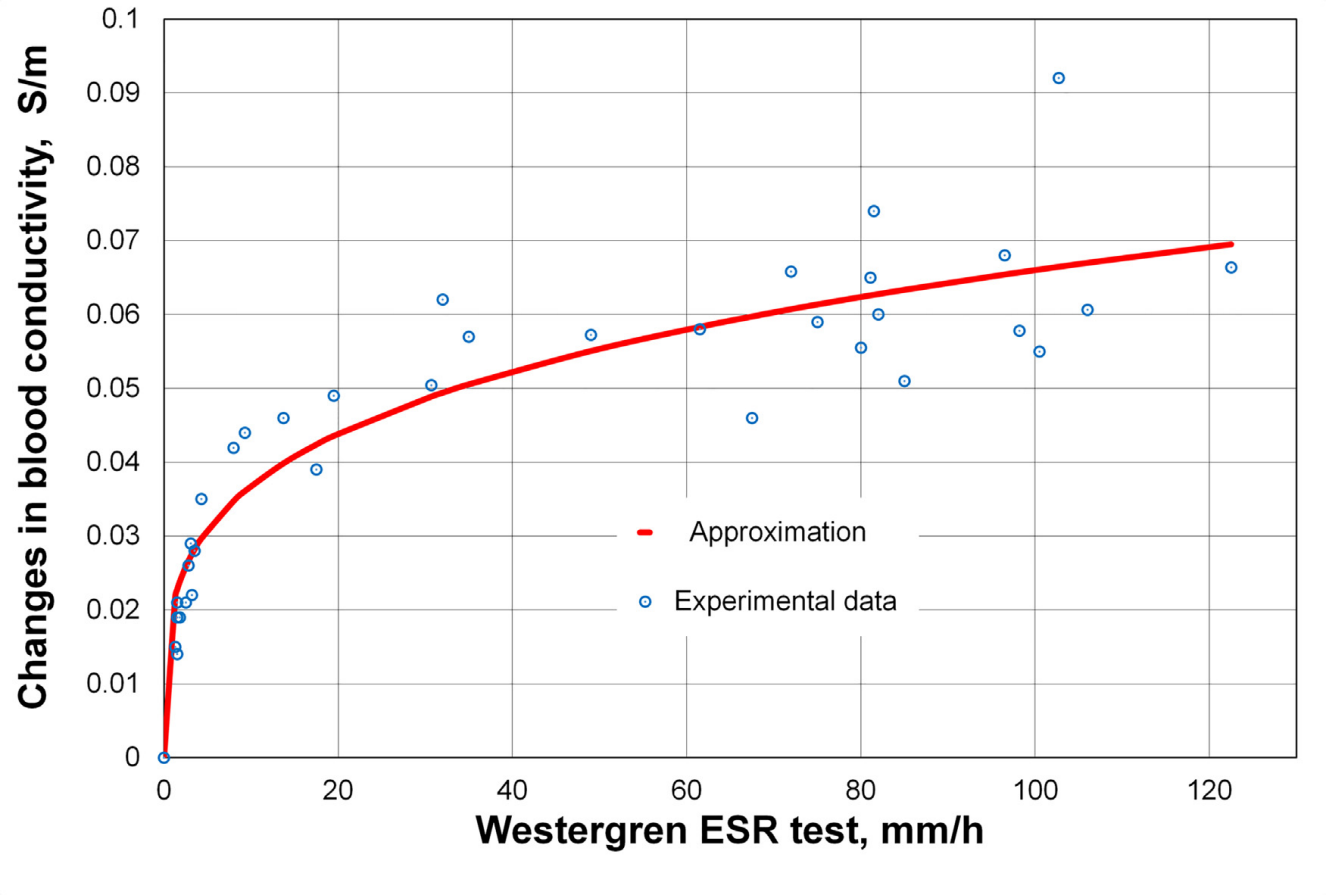
Correlation between 60 min Westergren ESR test and rate of changes in blood conductivity. EDTA-treated blood samples were prepared at different HCT and concentration of dextran or PBS. Each blood sample was loaded into the PDMS chamber and the Westergren tube at the same time.

We studied 40 different samples of blood. Blood samples of various HCT were prepared by mixing the erythrocytes with pure plasma as well as by adding dextran or PBS at a certain concentration to cover all the possible ranges of the standard Westergren tube. The HCT of each sample was between 35 and 55% as in a real human blood. We tested various factors to describe the correlation between our method and 60 min Westergren ESR test. We decided to use the difference in measured blood conductivity at 200 s and 400 s of recording. This difference defines the initial rate of the decrease in blood conductivity. We took 200 s as a starting point to avoid the influence of initial erythrocyte aggregation and instability. Fig 9 presents our experimental data and the approximation curve.

The used approximation is:

$$\Delta \sigma =\lambda W^{\gamma },$$ (23)

where Δ*σ* is the difference in blood conductivity (*σ*_200_-*σ*_400_) in S/m; *W* is Westergren rate in mm/h; *λ* and *γ* are fitting parameters; and *σ*_200_ and *σ*_400_ are conductivities at 200 and 400 s of sedimentation, respectively. The fitting parameters were calculated by least square method: *λ* = 0.02049 and *γ* = 0.254. The minimal sum of squared residuals was:

$$S=\sum_{i=1}^{N}{\left[{\left(\Delta \sigma \right)}_{i}-\lambda W_{i}^{\gamma }\right]}^{2}=0.0021\;{(\text{S/m)}}^{2},$$ (24)

where *N* is the number of experimental data. The average difference between the approximating curve and measured value of the difference in blood conductivity was:

$$\bar{\Delta }=\frac{1}{N}\sum_{i=1}^{N}\left|{\left(\Delta \sigma \right)}_{i}-\lambda W_{i}^{\gamma }\right|=0.0058\;\text{S/m}.$$ (25)

It should be noted that the dextran of high molecular weight increases the resistivity and viscosity of the blood sample. Also note that the behavior of cell aggregation differs in whole blood and under the influence of dextran. Nevertheless, Fig 9 shows that the correlation between our method of recording the changes in blood conductivity and the standard Westergren method is clearly seen. We hope to confirm the correlation in the clinical setting as, for example, in the work of Shteinshnaider *et al*. [11] where the authors studied 220 randomly chosen adult patients hospitalized in a medical department for various disorders.

## Conclusions

In this study, we investigated changes in blood conductivity during aggregation and sedimentation, using a system consisting of a small chamber with two planar electrodes on the bottom. As measured experimentally, the conductivity of blood in the chamber increased slightly during the first minute of observation and decreased thereafter for more than 1.5 h. We hypothesized that the slight increase in the blood conductivity within the first minute is due to erythrocyte aggregation.

To study this effect, we developed a theoretical model for calculating conductivity, in which an erythrocyte was modeled as a conducting oblate spheroid coated with a thin insulating membrane. Subsequently, we considered the conductivity of erythrocyte rouleaux formations, modeling the rouleaux as prolate spheroids with three components of conductivity. Dielectric theory was applied to determine the conductivity of the whole blood. Our theoretical calculations predict that the conductivity increases with the number of erythrocytes that have aggregated, and these calculations were found to be in good agreement with experimental data. Conductivity saturates when the number of erythrocytes in aggregates exceeds two dozen. Thus, we can say that, a short time after the beginning of sedimentation, blood conductivity depends only on the local volume fraction of erythrocytes.

We have provided additional experimental support for our hypothesis that the increase in the conductivity of blood in the initial stage of sedimentation is caused by the aggregation of red blood cells. We elucidated the effects of red blood cell aggregation by suspending the cells in PBS. In this case, the conductivity does not increase in the initial stage. Further, we mixed dextran of high molecular weight with whole blood to enhance aggregation. In this case, a very sharp increase in conductivity takes a very short time. The subsequent decrease in conductivity that was observed in our experimental data resulted from the deposition of red blood cells at the bottom of the container.

We used capillary tubes to determine the sedimentation curve for whole blood and the change in the velocity of sedimentation with time. Theoretical estimates of the sedimentation velocities were found to be in good agreement with the experimental data. As is well established, the erythrocyte sedimentation rate may be described by a sigmoid shaped curve. We have proved that this shape also arises for small thin tubes. Additionally, we experimentally evaluated the profile of the cell volume fraction, and proposed a simple model of erythrocyte sedimentation. As a result, we were able to describe the relationship between ESR and blood conductivity. The measured conductivity of blood is significantly reduced at the initial stage of deposition, and our method is very sensitive at the initial period of observation. We have experimentally shown a correlation between our method of recording the changes in blood conductivity and the standard Westergren method. This allows us to hope that our method will ultimately become applicable in clinical practice, as a means of decreasing the time required for ESR tests.

In the future, it would be interesting and important to have independent experimental verification of the correlation between the aggregate sizes and the blood conductivity. We plan to change the shape of the erythrocytes from biconcave discs to flat discs, ellipsoids, and spheres by varying osmotic pressure. This investigation should allow us to verify our theory and understand how cell shape influences aggregation and sedimentation.

## Supporting Information

S1 Fig Theoretical model of the suspension of randomly oriented spheroidal particles. Suspension of homogeneous spheroids in a homogeneous conducting medium.
(TIF)

S2 Fig Comparison of different effective medium theories and the experimental data. Conductivities at various hematocrits (HCTs) were calculated using a range of methods based on effective medium theory. Our experimental conductivity measurements are shown for comparison.
(TIF)

S1 Text The morphology of erythrocytes and the electrical properties of blood (review). In this text, we provide a review of the experimental and theoretical data relating to erythrocyte size and the electrical properties of human blood.
(DOC)

S2 Text The effective medium theory, theoretical background. We provide a review of the use of effective medium theory to describe the electrical conductivity of dilute particle suspensions. We compare different effective medium theories and the experimental data.
(DOC)
