# Supplementary figures and images for: Effects of Aggregation on Blood Sedimentation and Conductivity
Source: PLoS One. 2015 Jun 5;10(6):e0129337. doi: 10.1371/journal.pone.0129337 (PMC4457804; doi:10.1371/journal.pone.0129337)

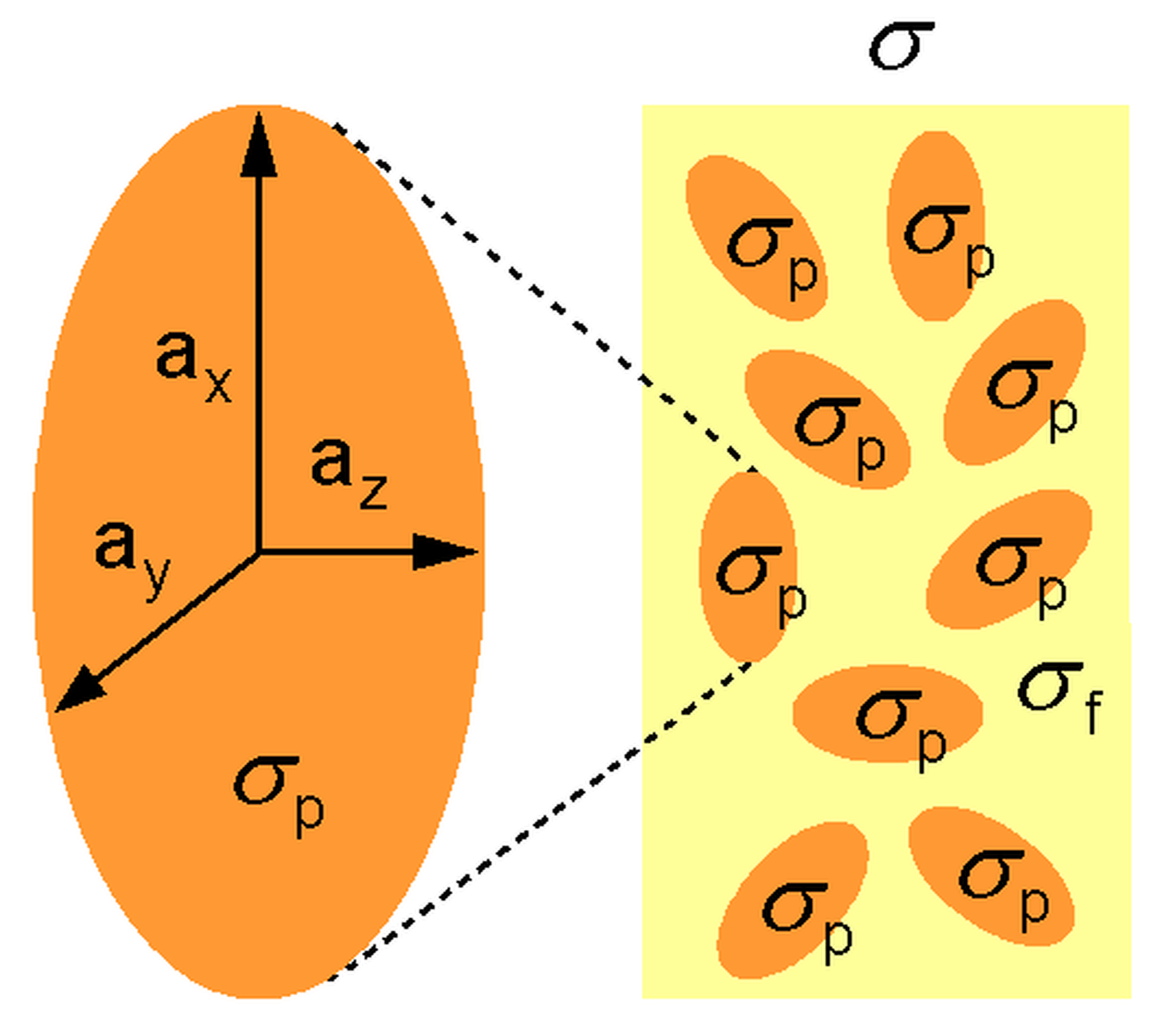

Supplement: S1 Fig — Suspension of homogeneous spheroids in a homogeneous conducting medium. (TIF) [file pone.0129337.s001.tif]

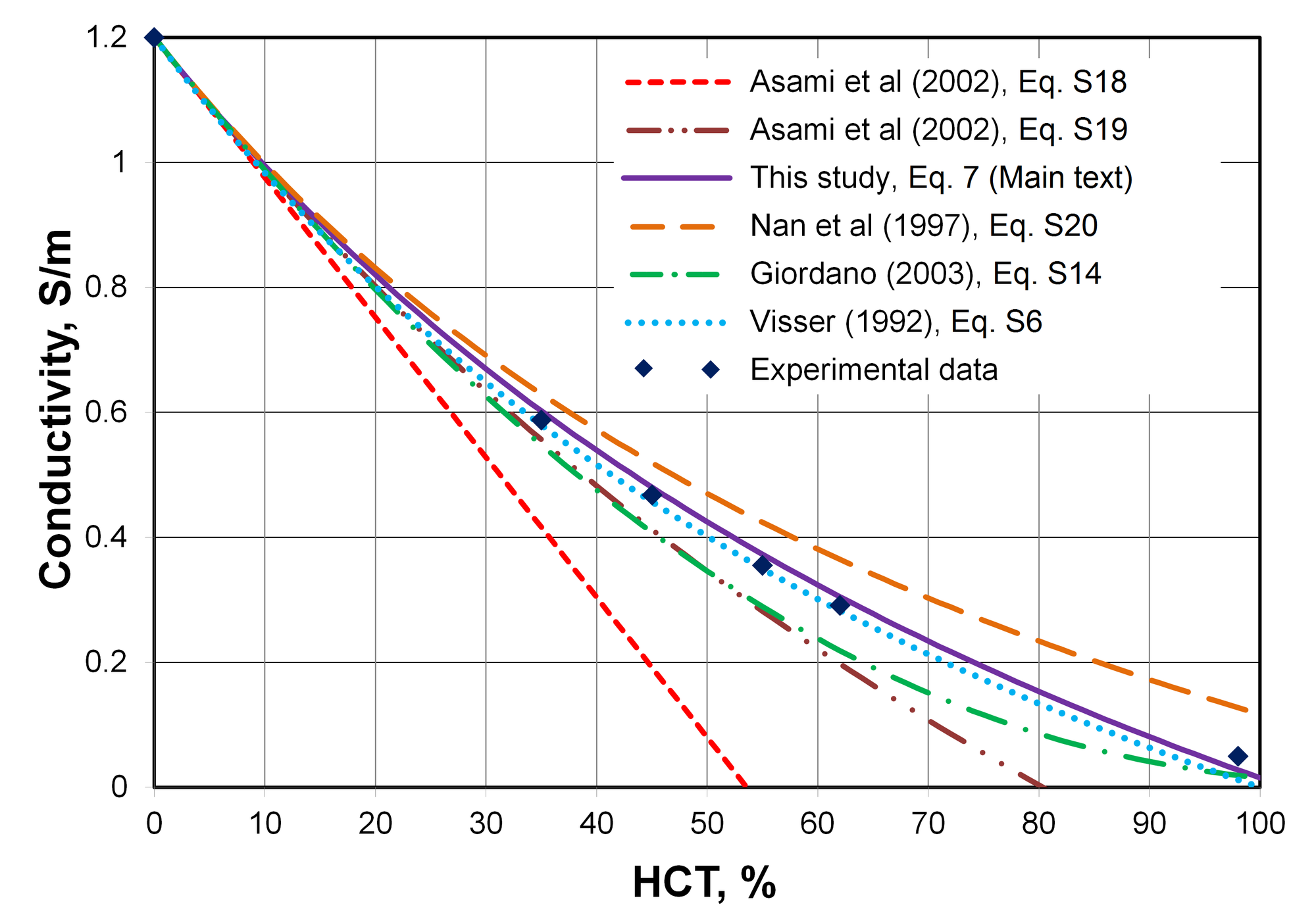

Supplement: S2 Fig — Conductivities at various hematocrits (HCTs) were calculated using a range of methods based on effective medium theory. Our experimental conductivity measurements are shown for comparison. (TIF) [file pone.0129337.s002.tif]
