# Supplementary material for: Effects of Aggregation on Blood Sedimentation and Conductivity
Source: PLoS One. 2015 Jun 5;10(6):e0129337. doi: 10.1371/journal.pone.0129337 (PMC4457804; doi:10.1371/journal.pone.0129337)
Supplement: S1 Text — In this text, we provide a review of the experimental and theoretical data relating to erythrocyte size and the electrical properties of human blood. (DOC) [file pone.0129337.s003.doc]

**Supporting Information Text S1**

**The morphology of erythrocytes and the electrical properties of blood (review)**

**Morphology of the erythrocyte**

At normal osmotic pressure, the human erythrocyte is a biconcave discoid with a diameter of approximately 8 μm, a minimum thickness of 1 μm, and a maximum thickness of 2.4 μm. It is surrounded by a thin insulating membrane. The experimental data relating to erythrocyte size are summarized in Table S1.

**Table S1. Dimensions of human erythrocyte derived from experime**ntal observations.

| **Diameter *D_ERY_* [*μ*m]** | **Minimum thickness *Δ* [*μ*m]** | **Average thickness *Δ* [*μ*m]** | **Maximum thickness *Δ* [*μ*m]** | **Volume *V_ERY_* [*μ*m^3^]** | **Membrane thickness δ [nm]** | **References** |
| --- | --- | --- | --- | --- | --- | --- |
| **8.28±0.10** |  | 1.71±0.06 |  | 82±2.7 |  | Houchin *et al.* (1958) [S1] |
|  |  |  |  |  | 6–7 | Hoffman (1962) [S2] |
| **7.3±0.3** |  |  |  |  |  | Coleman (1967) [S3] |
| **8.07±0.55** |  |  |  | 107.5±16.8 |  | Canham & Burton (1968) [S4] |
| **8.5±0.4** |  | 1.7±0.2 |  | 87 |  | Ponder (1971) [S5] |
| **7.82±0.62** | 0.81±0.35 | 1.70 | 2.58±0.27 | 94±14 |  | **Evans & Fung (1972) [S6]** |
|  |  |  |  |  | 7.8 | Hochmuth *et al.* (1983) [S7] |
| **6–9** |  |  |  | 80–100 |  | Sethu *et al.* (2006) [S8] |
| **8.2±0.4** |  |  | 2.6±0.2 | 89*.*1±1*.*8 |  | Livshits *et al.* (2007) [S9] |

The simplest model of an erythrocyte is a sphere covered with a thin shell. A sphere of volume equivalent to an erythrocyte (90 μm^3^) has a diameter of *D_ERY_* = 5.56 μm. Alternative models for erythrocytes are presented in Table S2. In the present study, we modeled erythrocytes as oblate ellipsoids encased by thin membranes.

**Table S**2. Theoretical models of a human erythrocyte.

| **Diameter**  ***D_ERY_* [*μ*m]** | **Maximum**  **thickness Δ [*μ*m]** | **Minimum**  **thickness Δ [*μ*m]** | **Membrane**  **thickness δ [nm]** | **Model** | **References** |
| --- | --- | --- | --- | --- | --- |
| **8.2** | 2.4 |  | 7.5 | Oblate ellipsoid | Bordi *et al.* (1997) [S10] |
| **8** | 2.2 | 1 | 7 | Degree-4 equation | Liu *et al.* (2003) [S11] |
| **7.8** | 2.5 |  |  | Oval of Cassini | Di Biasio and Cametti (2005)[S12] |
| **8** | 2 |  | 5 | Disc | Asami and Sekine (2007) [S13] |

**Electrical properties of human blood**

Dielectric spectroscopy is widely used to measure the dielectric properties of blood as a function of frequency. As deduced from a range of dielectric spectroscopy measurements, the electrical conductivities of erythrocyte membrane, cytoplasm, and blood plasma are shown in Table S3. Structurally, the erythrocyte membrane consists of two layers: the lipid bilayer, which contains functional proteins, and the membrane skeleton. However, it is very difficult to study this structure and to measure the dielectric constants of its different parts. Therefore, we assumed that the membrane had a uniform conductivity.

**Table S3. Dielectric properties of human blood derived from experimental observations.**

Passive electrical conductivities of the erythrocyte cell membrane *_m_*, erythrocyte cytoplasm *σ_cp_*, and blood plasma *σ_f_*.

| **Erythrocytes in solutions** | **T [°C]** | **σ*_m_* [S/m]** | ***σ_cp_* [S/m]** | ***σ_f_* [S/m]** | **References** |
| --- | --- | --- | --- | --- | --- |
| **Saline solutions** | 25 |  | 0.458 | 1.473 | Beving *et al.* (1994) [S14] |
| **Whole blood** | 25 |  | 0.458 | 1.22 | Beving *et al.* (1994) [S14] |
| **0.15 M NaCl** | 25 |  | 0.43·*σ_f_* |  | Bordi *et al.* (1990) [S15] |
| **0.15 M KCl** | 25 |  | 0.43·*σ_f_* |  | Bordi *et al.* (1990) [S15] |
| **0.15 M CsCl** | 25 |  | 0.36·*σ_f_* |  | Bordi *et al.* (1990) [S15] |
| **0.15 M LiCl** | 25 |  | 0.72·*σ_f_* |  | Bordi *et al.* (1990) [S15] |
| **0.15 M NaCl** | 37 | 3.5·10^–5^ | 0.675 | 1.85 | Diociaiuti *et al*. (1991) [S16] |
| **0.15 M NaCl** | 15 | 4.9·10^–5^ | 0.50 | 1.15 | Ballario *et al.* (1984) [S17] |
| **0.15 M NaCl** | 25 | 6.1·10^–5^ | 0.65 | 1.40 | Ballario *et al.* (1984) [S17] |
| **0.15 M NaCl** | 35 | 6.9·10^–5^ | 0.75 | 1.64 | Ballario *et al.* (1984) [S17] |
| **Whole blood** | 3.7 | 1·10^–6^ | 0.8 | 1.65 | Bordi *et al.* (1997) [S10] |

**References**

1. Houchin DH, Munn JI, Parnell BL. A method for the measurement of red cell dimensions and calculation of mean corpuscular volume and surface area. Blood, 1958; 13: 1185–1191.
2. Hoffman JF. Cation transport and structure of the red-cell plasma membrane. Circulation. 1962; 26: 1201–1213.
3. Coleman PN. Measurement of red cell diameter by image shearing. J Clin Path. 1967; 20: 915–917.
4. Canham PB, Burton AC. Distribution of size and shape in populations of normal human red cells. Circ Res. 1968; 22: 405–422.
5. Ponder E. Hemolysis and related phenomena, Chap II: Shape changes unaccompanied by volume changes. New York: Grune and Stratton; 1971.
6. **Evans E, Fung YC.** Improved measurements of the erythrocyte geometry. Microvasc Res. 1972; 4: 335–347.
7. Hochmuth RM, Evans EA, Wiles HC, McCown JT. Mechanical Measurement of Red Cell Membrane Thickness. Science. 1983; 220: 101–102.
8. Sethu P, Sin A, Toner M. Microfluidic diffusive filter for apheresis (leukapheresis). Lab Chip. 2006; 6: 83–89.
9. Livshits L, Caduff A, Talary MS, Feldman Y. Dielectric response of biconcave erythrocyte membranes to D- and L-glucose. J Phys D-Appl Phys. 2007; 40: 15–19.
10. Bordi F,·Cametti C, Misasi R, De Persio R, Zimatore G. Conductometric properties of human erythrocyte membranes: dependence on haematocrit and alkali metal ions of the suspending medium. Eur Biophys J. 1997; 26: 215–225.
11. Liu C, Sheen D, Huang K. A Hybrid Numerical Method to Compute Erythrocyte TMP in Low-Frequency Electric Fields. IEEE Trans Nanobiosci.2003; 2: 104–109.
12. Di Biasio A, Cametti C. Effect of the shape of human erythrocytes on the evaluation of the passive electrical properties of the cell membrane. Bioelectrochemistry. 2005; 65: 163–169.
13. Asami K, Sekine K. Dielectric modelling of erythrocyte aggregation in blood. J Phys D-Appl Phys, 2007; 40: 2197–2204.
14. Beving H, Eriksson LEG, Davey CL, Kell DB. Dielectric properties of human blood and erythrocytes at radio frequencies (0.2-10 MHz); dependence on cell volume fraction and medium composition. Eur Biophys J. 1994; 23: 207–215.
15. Bordi F, Cametti C, Di Biasio A. Determination of cell membrane passive electrical properties using frequency domain dielectric spectroscopy technique. A new approach. Biochim Biophys Acta-Biomembr. 1990; 1028: 201–204.
16. Diociaiuti M, Molinari A, Calcabrini A, Arancia G, Isacchi G, et al. Alteration of the passive electrical properties of adriamycin-treated red cell membrane deduced from dielectric spectroscopy. Bioelectrochem Bioenerg, 1991; 26: 177–192.
17. Ballario C, Bonincontro A, Cametti C, Rosi A, Sportelli L. Effect of extracellular alkali metal salts on the electric parameters of human erythrocytes in normal and pathological conditions. Z Naturforsch (C). 1984; 39: 1163–1169.
