# Supplementary material for: Effects of Aggregation on Blood Sedimentation and Conductivity
Source: PLoS One. 2015 Jun 5;10(6):e0129337. doi: 10.1371/journal.pone.0129337 (PMC4457804; doi:10.1371/journal.pone.0129337)
Supplement: S2 Text — We provide a review of the use of effective medium theory to describe the electrical conductivity of dilute particle suspensions. We compare different effective medium theories and the experimental data. (DOC) [file pone.0129337.s004.doc]

**Supporting Information Text S2**

**The effective medium theory, theoretical background**

**The Maxwell formula**

We have based our method for calculating the effective conductivity of whole blood on effective medium theory. The effective medium theory for dispersions of dielectric particles was proposed by Maxwell in 1873 [S1]. The Maxwell formula for a strongly diluted suspension of spheres in three-dimensional space is:

, (S1)

where *σ_f_* is the conductivity of the suspending medium (fluid), *σ_p_* is the conductivity of the embedded spherical particles, *φ_p_* is the volume fraction of the particles, and *σ* is the equivalent conductivity of the mixture.

**Fricke Theory and the Maxwell-Fricke equation**

It seems more accurate to consider blood as a mixture of randomly oriented spheroidal particles (Figure S1). We define the axes of the ellipsoids to be *a_x_*, *a_y_*, and *a_z_* (aligned with the *x*, *y*, and *z* axes of the reference frame), in order to model spheroidal inclusions. In Fricke’s first paper (1924) [2], which was devoted to the study of the conductivity of a suspension of homogeneous spheroids in a homogeneous conducting medium, he expressed his results for ellipsoids in the form:

, (S2)

where *C* is a factor that depends on the geometry and orientation of the inclusion.

For a random orientation of ellipsoids, *C* is given by:

$C=\frac{1}{3}\left[\frac{4}{2+(\sigma_{p}/\sigma_{f}-1)M}+\frac{1}{1+(\sigma_{p}/\sigma_{f}-1)(1-M)}\right]\left(1-\frac{\sigma_{p}}{\sigma_{f}}\right)$ . (S3)

The dimensionless parameter *M* is given by:

(S4)

for oblate ellipsoid shaped particles, where cos*ϕ* = *a_z_*/*a_x_* and the axes of the ellipsoid are *a_x_* = *a_y_* and *a_z_*. For oblate ellipsoids, *M* has a value between 0 (*a_z_*/*a_x_* = 0) and 2/3 (*a_z_*/*a_x_* = 1). For prolate ellipsoids:

, (S5)

where cos*ϕ*' = *a_x_*/*a_z_*.

For human blood, the conductivity of erythrocytes is negligible in comparison with that of plasma, *σ_p_* ≈ 0. This leads to Maxwell-Fricke equation:

$\sigma=\frac{\sigma_{f}}{1+\frac{H}{100-H}C}$ , (S6)

where *H* is the hematocrit in per cent and *C* is a constant defined by Eq. S3. Despite its simplicity, Eq. S6 has been used in many studies and demonstrates good agreement with experimental data [S3-S7].

**Extension of the effective medium theory to concentrated suspensions**

In the previous subsection we have described Maxwell’s relation and Fricke’s expressions for the electrical conductivity of dilute particle suspensions. The Bruggeman effective medium theory [S8] can be applied to the case of high volume fractions.

Let us consider the ellipsoid with semi-principal axes of length *a_x_*, *a_y_*, and *a_z_*. Equation S7 defines the auxiliary function *R*:

$R(s)=\sqrt{(s+a_{x}^{2})(s+a_{y}^{2})(s+a_{z}^{2})}$ . (S7)

The depolarization factors (*L*) along each axis are given by:

$L_{x}=\frac{a}{2}\int_{0}^{+\infty} \frac{\mathrm{d}s}{(s+a_{x}^{2})R(s)};L_{y}=\frac{a}{2}\int_{0}^{+\infty} \frac{\mathrm{d}s}{(s+a_{y}^{2})R(s)};L_{z}=\frac{a}{2}\int_{0}^{+\infty} \frac{\mathrm{d}s}{(s+a_{z}^{2})R(s)}$ , (S8)

in which *L_x_* + *L_y_* + *L_z_* = 1, *a* = *a_x_a_y_a_z_*. The depolarization factor is the ratio of the polarizability of the inclusion to the polarizability of the medium as a whole. These factors depend only on the geometrical parameters of the ellipsoid. For example, in the case of a thin disc *L_x_* = *L_y_* = 1/2, *L_z_* = 0 and in the case of a sphere *L_x_* = *L_y_* = *L_z_* = 1/3.

We model blood as a mixture of rotational ellipsoid inclusions. We can therefore assume that *a_x_* = *a_y_* and consequently define the axis ratio as *ξ* = *a_z_*/*a_x_* = *a_z_*/*a_y_*. Prolate ellipsoids (of pointy or elongated form) have *ξ* > 1, whereas oblate ellipsoids (of planetary or flattened form) have *ξ* <1. After substituting the expression for axis ratio *ξ* into the integrals Eq. S8, the depolarization factors may be expressed in closed form as shown in Eq. S9 and Eq. S10. The value of each factor depends on the shape of the ellipsoids:

$L_{x}=L_{y}=\frac{\xi}{2}\int_{0}^{+\infty} \frac{\mathrm{d}\zeta}{(\zeta+1)^{2}(\zeta+\xi^{2})^{1/2}}=\left\{\begin{matrix} \frac{\xi}{4p^{3}}\left[2\xi p+\ln\frac{\xi-p}{\xi+p}\right] & \mathrm{if} \xi>1, \\ \frac{\xi}{4q^{3}}\left[\pi-2\xi q-2\arctan\frac{\xi}{q}\right] & \mathrm{if} \xi<1, \end{matrix}\right.$ (S9)

$L_{z}=\frac{\xi}{2}\int_{0}^{+\infty} \frac{\mathrm{d}\zeta}{(\zeta+1)(\zeta+\xi^{2})^{3/2}}=\left\{\begin{matrix} \frac{1}{2p^{3}}\left[\xi\ln\frac{\xi+p}{\xi-p}-2p\right] & \mathrm{if} \xi>1, \\ \frac{1}{2q^{3}}\left[2q-\xi\pi+2\xi\arctan\frac{\xi}{q}\right] & \mathrm{if} \xi<1, \end{matrix}\right.$ (S10)

where $p=\sqrt{\xi^{2}-1}$ and $q=\sqrt{1-\xi^{2}}$. Appling Bruggeman’s procedure [S8] to a dispersion of ellipsoids, one can derive the resulting relationships for effective conductivity. The following relations have been derived by many authors [S9, S10]. For small concentrations of erythrocytes (*φ_p_* << 1) [S9, S10]:

. (S11)

According to Giordano [S10], the equivalent conductivity of a mixture *σ* with a volume fraction *φ_p_* < 0.1 is:

. (S12)

Asami [S9] proposed a different relation for these suspensions (*φ_p_* < 0.1):

. (S13)

For large concentrations of erythrocytes (*φ_p_* < 1), the equation of equivalent conductivity takes the following form [S9, S10]:

, (S14)

where *L* = *L_x_* = *L_y_*, *L_z_* = 1 – 2 *L* . For spherical inclusions, *L* = 1/3 and Eq. S14 reduces to:

. (S15)

This is the Bruggeman equation for dispersions of spheres that are not dense.

**Conductivity of whole blood**

We propose a model for calculating the electrical conductivity of blood that is based on Eq. S12, but incorporates a more detailed representation of erythrocytes. In fact, the typical erythrocyte of a healthy human is a biconcave discoid encased by a thin insulating membrane. The conductivity of the cytoplasm inside the erythrocyte is significantly higher than that of the membrane. To represent the erythrocytes, we employ a shell-ellipsoid model in which the internal ellipsoid corresponds to the cytoplasm and the external confocal ellipsoid includes both the cytoplasm and the membrane. The thickness of the erythrocyte membrane *δ* is very small compared with the erythrocyte radius, *R_ERY_*, and its thickness, *Δ* (see Figure 3B). Hence, *δ/a_x_, δ/a_y_,* and *δ/a_z_* << 1, and the volume ratio *η* of the inner ellipsoid to the outer ellipsoid may be approximated by:

$\eta\approx\left(1-\frac{\delta}{a_{x}}\right)\left(1-\frac{\delta}{a_{y}}\right)\left(1-\frac{\delta}{a_{z}}\right)$ . (S16)

The equivalent conductivity of a shell-ellipsoid is a tensor that has three components along the *x*-, *y*- and *z*-axis of the ellipsoid: *σ_px_*, *σ*_p_*_y_*, and *σ_pz_*, respectively. For an axis *k* (*k* = *x, y, z*), the component *σ_pk_* can be expressed as [S9]:

$\sigma_{pk}=\sigma_{m}\frac{\beta_{k}\sigma_{m}+\sigma_{cp}-\beta_{k}\eta(\sigma_{m}-\sigma_{cp})}{\beta_{k}\sigma_{m}+\sigma_{cp}+\eta(\sigma_{m}-\sigma_{cp})}$ , (S17)

where *σ_m_* and *σ_cp_* are the electrical conductivity of the membrane and cell cytoplasm, respectively, and *β_k_* = (1 ‑ *L_k_*)/*L_k_*. In this work, we assume that the erythrocytes have random orientations and are randomly distributed in the plasma. Thus, the effective conductivity of the blood is a scalar, despite the fact that the conductivity of each cell is a tensor.

When shell-ellipsoids are dispersed at a small volume fraction *φ_p_* in a continuous medium of conductivity *σ_f_*, the conductivity *σ* of the suspension (*φ_p_* << 1) is given by [S9]:

. (S18)

For concentrations where *φ_p_* < 0.1, the effective conductivity can be expressed as [S9]:

. (S19)

Nan *et al.* [S11] proposed an equation for randomly oriented ellipsoidal particles:

, where $\alpha_{k}=\frac{\sigma_{pk}-\sigma_{f}}{\sigma_{f}+L_{k}(\sigma_{pk}-\sigma_{f})}$ . (S20)

Thus, we have discussed the theoretical methods for calculating the effective conductivity of heterogeneous mixtures. The theory developed in two directions: toward increasing concentrations of the suspensions and toward more complex forms of inclusions. Strongly diluted suspensions were studied in earlier theories. Diluted suspensions (volume fraction *φ_p_* << 1) and concentrated suspension (*φ_p_* < 0.1) were considered later. With increasing complexity, the following inclusion shapes were considered: sphere, ellipsoid, and shell-ellipsoid.

**Comparison of theoretical and experimental data for the effective conductivity**

Figure S2 presents our experimental and theoretical result for the change in blood conductivity with HCT. The theoretical results of other authors are shown in Supporting Information for comparison. We assumed a mixture of disaggregated erythrocytes in the shape of oblate spheroids with *a_x_* = *a_y_* = *R_ERY_* and *a_z_* = *Δ* / 2. Using Eq. S9 and Eq. S10, we calculated the depolarization factors *L_x_* = *L_y_* = 0.1482 and *L_z_* = 0.7036. The principal conductivities given by Eq. S17 were *σ_px_* = *σ_py_* = 0.0281 S/m and *σ_pz_* = 0.00623 S/m.

The red dotted line in Figure S2 corresponds to Eq. S18 [S9]. Equation S11 [S9, S10] produces the same curve at a conductivity *σ_p_* = 0.0153 S/m. The brown dashed-dotted-dotted line was obtained from Eq. S19 [S9]. Note that Eq. S11, Eq. S18, and Eq. S19 show nonphysical behavior at high HCT. The orange long-dashed line shows Eq. S20 [S11]. Compared with our experimental results (dark blue rhombuses), this equation overestimates the conductivity at high HCT. The green dashed-dotted line is the solution of the nonlinear Eq. S14 [S9, S10]. The blue dotted line corresponds to Eq. S6 [S7]. This equation agrees very well with our experimental data, but it is worth noting that the factor *C* was not calculated analytically according to Eq. S3. Instead, this factor was treated as a fitting parameter, resulting in the value *C* = 1.987 [S7]. Apparently, it contains information about the shape of the erythrocyte and the conductivities of its components.

In the future, we plan to change the osmotic pressure and to examine the sedimentation of erythrocytes that have a modified shape. Therefore, we prefer to use a formula that allows such changes to be incorporated, such as Eq. 7. Further, we applied this Eq. 7 to calculate the conductivity of rouleaux, as described later in this article. Finally, the conductivities calculated using our theoretical method [solid violet line, based on Eq. 7] show good agreement with our experimental data in Figure ~~4~~. If we use a value of σ*_p_* = 0.0153 S/m for the conductivity of embedded spheroids and apply the model of randomly oriented spheroidal particles, then Giordano’s [46] Eq. S12 provides almost exactly the same values as our Eq. 7. We have therefore concluded that, for randomly oriented particles, there is no significant difference between a simple ellipsoidal inclusion model and the model of ellipsoids encased in a thin shell.

**References**

1. Maxwell JC. A Treatise on Electricity and Magnetism. Oxford: Clarendon Press; 1873.
2. Fricke H. A mathematical treatment of the electric conductivity and capacity of disperse systems. I. The electric conductivity of a suspension of homogeneous spheroids. Phys Rev. 1924; 24: 575–587.
3. Zhao TX, Lockner D. Electrical impedance and erythrocyte sedimentation rate (ESR) of blood. Biochim Biophys Acta-Biomembr. 1993; 1153: 243–248.
4. Hill DW, Thompson FD. The effect of haematocrit on the resistivity of human blood at 37 degrees C and 100 kHz. Med Biol Eng. 1975; 13: 182–186.
5. Mohapatra SN, Hill DW. The Changes in Blood Resistivity with Haematocrit and Temperature. Intensive Care Med. 1975; 1: 153–162.
6. Sandberg K, Sjöqvist BA, Olsson T. Relation between Blood Resistivity and Hematocrit in Fresh Human Fetal Blood. Pediatr Res, 1981; 15: 964–966.
7. Visser KR. Electric conductivity of stationary and flowing human blood at low frequencies. Med Biol Eng Comput. 1992; 30: 636–640.
8. Bruggeman DAG. Berechnung verschiedener physikalischer Konstanten von heterogenen Substanzen. I. Dielektrizitätskonstanten und Leitfähigkeiten der Mischkörper aus isotropen Substanzen. Ann Phys-Berlin, 1935; 416: 636–664.
9. Asami K. Characterization of heterogeneous systems by dielectric spectroscopy. Prog Polym Sci. 2002; 27: 1617–1659.
10. Giordano S. Effective medium theory for dispersions of dielectric ellipsoids. J Electrostat. 2003; 58: 59–76.
11. Nan CW, Birringer R, Clarke DR, Gleiter H. Effective thermal conductivity of particulate composites with interfacial thermal resistance. J Appl Phys. 1997; 81: 6692–6699.
